# Trust in the health care professional and health outcome: A meta-analysis

**DOI:** 10.1371/journal.pone.0170988

**Published:** 2017-02-07

**Authors:** Johanna Birkhäuer, Jens Gaab, Joe Kossowsky, Sebastian Hasler, Peter Krummenacher, Christoph Werner, Heike Gerger

**Affiliations:** 1 Clinical Psychology and Psychotherapy, Department of Psychology, University of Basel, Basel, Switzerland; 2 Department of Anesthesiology, Perioperative and Pain Medicine, Boston Children’s Hospital/Harvard Medical School, Boston, Massachusetts, United States of America; 3 Program in Placebo Studies and the Therapeutic Encounter, Beth Israel Deaconess Medical Center, Harvard Medical School, Boston, Massachusetts, United States of America; 4 Collegium Helveticum, University of Zurich and ETH Zurich, Zurich, Switzerland; University of Marburg, GERMANY

## Abstract

**Objective:**

To examine whether patients’ trust in the health care professional is associated with health outcomes.

**Study selection:**

We searched 4 major electronic databases for studies that reported quantitative data on the association between trust in the health care professional and health outcome. We screened the full-texts of 400 publications and included 47 studies in our meta-analysis.

**Data extraction and data synthesis:**

We conducted random effects meta-analyses and meta-regressions and calculated correlation coefficients with corresponding 95% confidence intervals. Two interdependent researchers assessed the quality of the included studies using the Strengthening the Reporting of Observational Studies in Epidemiology guidelines.

**Results:**

Overall, we found a small to moderate correlation between trust and health outcomes (r = 0.24, 95% CI: 0.19–0.29). Subgroup analyses revealed a moderate correlation between trust and self-rated subjective health outcomes (r = 0.30, 0.24–0.35). Correlations between trust and objective (r = -0.02, -0.08–0.03) as well as observer-rated outcomes (r = 0.10, -0.16–0.36) were non-significant. Exploratory analyses showed a large correlation between trust and patient satisfaction and somewhat smaller correlations with health behaviours, quality of life and symptom severity. Heterogeneity was small to moderate across the analyses.

**Conclusions:**

From a clinical perspective, patients reported more beneficial health behaviours, less symptoms and higher quality of life and to be more satisfied with treatment when they had higher trust in their health care professional. There was evidence for upward bias in the summarized results. Prospective studies are required to deepen our understanding of the complex interplay between trust and health outcomes.

## Introduction

Patients’ trust in their health care professional is central to clinical practice [1, 2]. The General Medical Council states that "(p)atients must be able to trust doctors with their lives and health" and that maintaining trust is one core guidance for physicians [3]. Similar obligations are part of codes of conduct for other health care professionals such as nurses [4] or psychotherapists [5]. Patients have to trust their health care professionals to work in their best interest and outcome [6]. In this regard, trust in the health care professional has been suggested to be the foundation for effective treatments [7, 8] and fundamental for patient-centered care [9].

Besides such a deontological obligation for trust theoretical models describe mechanisms on how trust may influence health outcomes [7, 10–12]. Some of those conceptualize trust in the health care provider in relation to the patient-clinician relationship, which has previously been shown to be significantly associated with health outcomes across 13 RCTs [13]. Therefore, the question arises whether trust in the health care professional is as well associated with patients’ health.

Empirical evidence regarding this question comes from a growing number of studies that report correlations between trust measures and patients’ health outcome. In the different studies the health outcomes encompass different dimensions, such as objectively measured indicators (e.g. CD4 cell counts) [14], clinical observations (e.g. clinical diagnoses) [15], and patients’ subjective self-ratings (e.g. patient satisfaction) [16]. The association between trust and health outcome has been found to differ across individual studies. For instance,in a sample of patients with diabetes, trust in the health care professional was found to be positively related to objective and subjective health outcomes (glycemic control, health-related quality of life, and patient satisfaction) [12]. In contrast, there was no significant association between trust in the health care professional and subjective outcomes (blood pressure control) in patients with hypertension [17]. In the absence of a systematic and comprehensive summary of the available evidence the variation in the observed health outcomes and in disorders complicates conclusions regarding the association between trust and health.

The empirical confirmation of the assumed association between trust and health outcome would strengthen the—so far—ethically derived claims for trustful and patient-centered relationships in clinical settings [2, 18, 19]. Therefore, a comprehensive and differentiated summary of the available evidence is needed. We conducted a systematic review with meta-analysis of observational studies in order to (1) estimate the overall association between trust and health outcome and to (2) investigate whether the strength of such an association depends on the type of health outcome. We controlled for a potential impact of study methodology and design on the association between trust and health outcome.

## Methods

### Data sources and searches

**Fig 1:**
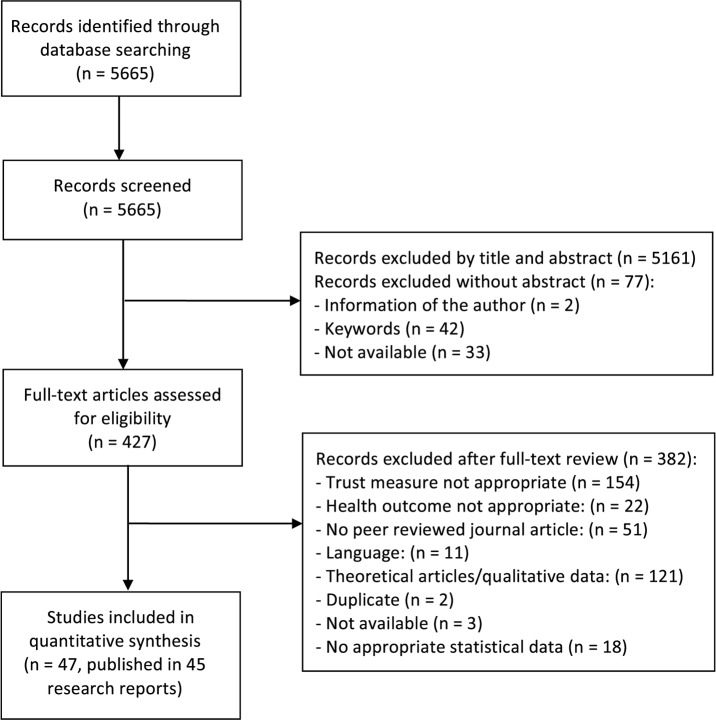
Study selection procedure.

We conducted a systematic literature search in bibliographic databases (CINAHL, Embase, MEDLINE and PsycINFO; see S1 File, which summarizes the applied search strategies). All records were transferred to EndNote (EndNote X7 Thomson Reuters, USA), where duplicates were eliminated and all titles and abstracts were screened for inclusion and exclusion criteria. Records were excluded if they clearly did not meet our inclusion criteria (J.B.). Two researchers then independently reviewed the full text of the records that were considered potentially relevant during title and abstract screening (J.B. and C.W.). Ambiguities were resolved by consensus or by consulting a third researcher (H.G.). If only the title, the author or journal names were provided by the electronic database search, we contacted the authors or searched the journal archives manually in order to check for eligibility. Records were excluded if we were unable to obtain the full-text (Fig 1).

### Study selection

We included studies that (1) reported quantitative data on the association between trust in the health care professional and health outcomes (exclusion of case vignettes, interviews, reviews, etc.), (2) took place in a health care setting, were written in English or German, (4) were published journal articles and (5) measured interpersonal trust (e.g. trust in the nurse, physician, GP, psychiatrist) with a valid, reliable and established trust questionnaire (i.e. included a reference to a published article which used the respective trust questionnaire; self-created, adapted and single-item questionnaires were excluded).

To qualify as health outcome the dependent variable needed to relate to at least one of the following health dimensions: (a) aetiology of the health problem (e.g. physiological measures, such as CD4 cell count, HbA1c), (b) symptoms (e.g. depression, worries, mood), (c) treatment-related indicators (e.g. adherence, health promoting lifestyle, satisfaction with treatment), or (d) consequences of being in treatment (e.g. patient satisfaction, health-related quality of life, functional level, overall health, cognitive and emotional change). In cases of uncertainty (e.g. online search behaviour) we required that the authors provide at least one reference to an article that showed an association between a particular outcome and one of the previously outlined health dimensions. Moreover, the outcome needed to have a clear direction in order to distinguish between positive versus negative health outcome (e.g. increase in CD4 cell count, and decrease on a scale measuring depression symptom severity indicate improvement). We excluded outcomes whenever it remained unclear whether an increase or decrease on the respective measure was a beneficial outcome for the patient (see S1 Table for a list of the included health outcomes).

### Data extraction and quality assessment

Two researchers (J.B. and S.H.) independently extracted correlations between trust in the health care provider and health outcomes whenever reported. If correlations were not reported, we used the available binary data (i.e. numbers / proportions of events) or the provided odds ratios in order to estimate the association between trust in the health care provider and health outcome. Binary data and odds ratios were transformed into log odds ratios, then into Fisher’s Z and finally into correlations. If data from two studies were reported in one publication, we extracted data from each study independently.

We extracted the diagnoses in the patient sample, the country in which the study was conducted, the duration of treatment and characteristics of the trust scale. For quality assessment of the included studies we used the STROBE (Strengthening the Reporting of Observational Studies in Epidemiology) checklist with a maximum of 22 points [20].

Health outcomes were clustered into objective (e.g. blood pressure, HbA1C, BMI), observer-rated (e.g. diagnosis by a professional) as well as self-rated subjective outcomes. The latter were divided into self-reported health behaviours (e.g. medication adherence, screening behaviour, health promoting lifestyle, online search behaviour) and health-related subjective experiences (e.g. patient satisfaction, health-related quality of life, pain-related anger, depression, worries).

Following a thorough training, data extraction and coding was conducted independently by two researchers (J.B. and S.H.) using a standardized form (Microsoft Office Excel 2007). Discrepancies between the two coders were resolved in face-to-face discussions or by consulting a third researcher (H.G.) when no consensus could be found.

### Data synthesis and analysis

We calculated correlation coefficients in order to estimate the association between trust in the health care professional and health outcome with the corresponding 95% confidence interval (CI). With regard to the magnitude of effect sizes, we interpreted correlation coefficients in the order of 0.10 as “small”, those of 0.30 as “moderate,” and those of 0.50 or higher as “large” [21]. Whenever data was reported for subgroups of study participants in one publication, we calculated the weighted mean correlation [22]. If the N was missing in the table of analysis, we used the N of the descriptive statistics, ignoring the possibility that not all study participants may have contributed data for the correlational analyses. If only subscales of a composite health outcome measure were reported, we coded the available subscale data according to our classification of health outcomes. However, we preferred total scores over subscale data, if available. When more than one health outcome was reported in a publication, we combined the data from different outcome measures so that each study contributed only one estimate per analysis to avoid dependencies in the data.

In a primary analysis, we calculated an overall estimate across all health outcomes. Then, we calculated separate estimates for objective, observer-rated and self-rated subjective outcomes. We further divided subjective self-rated outcomes into health behaviours and subjective experiences and estimated the impact of potential moderators (i.e. study quality, duration of the treatment, the country in which the study was conducted, and the applied trust questionnaire). In an exploratory sub-analysis we divided subjective experiences into patient satisfaction, quality of life and symptom-related outcomes and conducted individual subgroup-analyses. For the subgroup and moderator analyses including categorical predictors we conducted stratified meta-analyses; in case of continuous outcomes we conducted meta-regressions.

We explored the presence of small study bias and publication bias by assessing funnel plot asymmetry (i.e. whether negative or non-significant findings are missing) with a regression test [23]. We inspected the Egger’s regression coefficient rather than the Begg’s correlation, since the power for this test is higher [24]. We calculated a fail-safe N, which determines the number of unretrieved studies with a null-finding that would bring the pooled estimate to zero [25]. Finally, we applied the trim and fill method, which adjusts the association between trust in the health care professional and health outcome for missing studies using the random effects model and adjusting for studies missing at the left side of the mean [26]. We used Comprehensive Meta-Analysis, version 2.0 (available at www.meta-analysis.com) for all meta-analyses and subgroup analyses, and STAT 13.1 for the meta-regressions. We used a two-sided *P*-value to test for statistical significance.

We applied random effects rather than a fixed effect model, since the included studies were expected to be heterogonous in several respects. To evaluate heterogeneity between studies, we examined τ^2^, which is an estimate of the variance among true effect sizes. τ (square root of τ^2^) represents the standard deviation of the distribution underlying the included trials assumed to be a random sample. Higher τ^2^-values indicate greater variability between studies than would be expected by chance. Based on the definition of small, moderate, and large effect size estimates according to Cohen in 1988 [21] we interpreted τ^2^ as follows: τ^2^ = (0.2/2)2 = 0.01 was considered to represent low heterogeneity, τ^2^ = 0.06 [(0.5/2)2] moderate heterogeneity, and τ^2^ = 0.16 [(0.8/2)2] high heterogeneity between studies. As a measure of heterogeneity, τ^2^ has been shown to be independent of the number of studies and patients included in a meta-analysis (i.e. no increase with large numbers of studies or large sample sizes) [27].

## Results

### Descriptives of included studies

We included 47 studies that were published in 45 reports (see S1 Table for descriptive information and the references of the included studies) with 34 817 participants (median: 200 participants, range: 24 to 8392). The procedure of study selection, including reasons for exclusion after full-text review, is shown in Fig 1. Studies were conducted in Asia (2), Europe (6), North America (34), and Australia (2). Two studies did not specify the country, and one study was conducted in more than one country. In 24 studies trust was measured by the Trust in Physician Scale [28], six studies used the Wake Forest Trust Scale [29], four studies the Trust Scale of the Primary Care Assessment Survey [30], three studies applied the Trust Scale of the Illness Concept Scale [31], two used the Consumer Assessment of Healthcare Providers and Systems (CAHPS) Cultural Competence [32], and two used the Trust Scale of the Cologne Patient Questionnaire [33]. Six trust questionnaires were only used in one study (see S1 Table for details). 33 studies reported correlations between trust in the health care professional and health outcome and 15 studies reported binary data or odds ratios. Most studies did not report the order of assessment of trust and outcome. Four studies specified that the trust measurement preceded the outcome assessment. No study reported on a reverse order of data collection (i.e. outcome assessment preceded the trust assessment). If no information was given, we assumed that trust and outcome were assessed at the same time-point. 15 studies reported objective health outcomes; two studies reported observer-rated outcomes and 42 studies reported subjective self-rated outcomes. Most studies included participants with chronic and multiple health complaints (see S1 Table). Six studies defined the duration of treatment with a median of 1.7 months (range: 10 days to 5 years). Two studies reported a mean of 2.6 and 2.7 visits in the study sample. Study quality ranged from 7.5 to 18.5 STROBE points (median: 15 mean: 13.5). Studies were published between 1981 and 2013 (median: 2009).

### Association between trust and health outcome: Primary, secondary and exploratory analyses

Across all outcomes, we found a small to moderate correlation between trust and health outcome (r = 0.24, 95% CI: 0.19 to 0.29) based on all 47 studies (Table 1 and S2 File, which shows all forest plots). Heterogeneity between studies was low to moderate.

**Table 1:** Associations between Trust in the Health Care Professional and Health Outcome stratified according to the Outcome Dimension.

Analysis	*N* of studies (patients)	r	95% CI	*p*	τ^2^	Publication bias		
						Egger test (p)	Fail-safe N	Trim & Fill test
Overall	47 (34 817)	0.24	0.19, 0.29	<0.001	0.03	<0.001	9 328	0.24
Objective	15 (7 867)	-0.02	-0.08, 0.03	0.430	0.01	0.518	0	-0.02
Observer-rated	2 (706)	0.10	-0.16, 0.36	0.445	0.04	-	-	-
Subjective, self-rated	42 (30 943)	0.30	0.24, 0.35	<0.001	0.04	<0.001	10 532	0.30
Behaviour	21 (26 642)	0.14	0.10, 0.19	<0.001	0.01	0.010	1 857	0.14
Experience	29 (10 229)	0.37	0.27, 0.47	<0.001	0.09	0.226	23 39	0.37
Satisfaction	15 (5 141)	0.57	0.49, 0.64	<0.001	0.04	0.636	8 007	0.57
HRQoL	5 (1 816)	0.18	0.14, 0.22	<0.001	<0.01	0.134	93	0.17
Symptom-related	13 (4 285)	0.13	0.04, 0.22	0.004	0.02	0.333	263	0.13

*Note*. Study is used as the the unit of analysis. Ns of Subanalysis (objective, observer-rated and subjective, self-rated) do not add up to 34 817, since several studies included more than one outcome. r = correlation; CI = confidence interval; τ^2^ = variability between studies;— = no estimate provided due to small number of included studies; HRQoL = health-related quality of life.

Stratified analyses revealed small and non-significant correlations in case of objective outcomes and observer-rated outcomes (r = -0.02, -0.08 to 0.03 and r = 0.10, -0.16 to 0.36 respectively; see S2 File for the respective forest plots), as well as a moderate correlation with regard to subjective self-rated outcomes (r = 0.30, 0.24 to 0.35). Heterogeneity was small to moderate.

In a subgroup analysis, we found a small correlation (r = 0.14, 0.10 to 0.19) between trust and patients’ health behaviours and a moderate correlation between trust and health-related subjective experiences (r = 0.37, 0.27 to 0.47; Table 1). Our final stratification of the health-related subjective experiences showed a large association between trust and patient satisfaction (r = 0.57, 0.49 to 0.64) and small associations between trust and health-related quality of life (r = 0.18, 0.14 to 0.22) and symptom-related outcomes (r = 0.13, 0.04 to 0.22). Small to moderate between-study heterogeneity remained unexplained in most explorative analyses (Table 1).

### Publication bias

Despite a significant Egger’s regression test in the overall analysis as well as in the analysis using only studies with a health behaviour as outcome (*P* < 0.001), the fail-safe N and the trim-and-fill analyses indicate a small risk for publication bias (Table 1 and S3 File, which shows the funnel plots).

### Meta-regressions and subgroup analyses

Meta-regressions with study quality and treatment duration as predictors showed that the association between trust and outcome depended on study quality, with smaller associations in higher quality studies (Table 2 and S4 File, which shows the scatter plots of the meta-regressions). Furthermore, based on six reports of treatment duration, we found the correlation between trust and outcome to be independent of treatment duration (Table 2 and S4 File). In both meta-regressions one outlier study was identified. Repeated analyses excluding the respective outliers showed similar results as the initial analyses (see S4 File).

**Table 2:** Meta-Regressions of the Association between Trust in the Health Care Professional and Health Outcome according to Study Quality, Duration of Treatment.

Moderator	*N* of studies	B	95% CI	*p*	τ^2^
STROBE (study quality)	47	-0.033	-0.07, -0.003	0.074	0.06
Duration of treatment	6	-0.001	-0.01, 0.01	0.751	0.02

*Note*. N = number of studies included in the analysis; B = unstandardized regression coefficient from meta-regression; CI = confidence interval; τ^2^ = variability between studies for the intercept of the model only; STROBE = Strengthening the Reporting of Observational Studies in Epidemiology (study quality).

Stratified analyses showed some variation in associations between trust and outcome when studies were conducted in different geographical regions but no variation when different trust questionnaires were used (Table 3). Further analyses showed considerable differences when correlational data vs. binary data were reported and finally a meta-analysis restricted to the prospective studies showed a comparable correlation as the analysis including all studies (see Table 3 and S2 File, which shows the respective forest plots).

**Table 3:** Associations between Trust in the Health Care Professional and Health Outcome stratified according to the Geographic Region, Trust Questionnaire and Type of Data.

Subgroup Analysis	*N* of studies (patients)	r	95% CI	*p*	τ^2^
Country^a^					
Asia	2 (536)	0.13	0.10, 0.15	<0.001	<0.01
Australia	2 (665)	0.35	-0.31, 0.79	0.298	0.25
Europe	6 (848)	0.36	0.22, 0.48	<0.001	0.03
North America	34 (31 780)	0.22	0.16, 0.28	<0.001	0.04
Trust Questionnaire^a^					
Trust in Physician Scale^b^	24 (17 650)	0.27	0.19, 0.35	<0.001	0.04
Other Trust Questionnaires	23 (17 167)	0.19	0.13, 0.25	<0.001	0.02
Type of Data					
Correlational Data	33 (19492)	0.27	0.26, 0.28	<0.001	<0.01
Binary Data	15 (206867)	0.05	0.03, 0.08	<0.001	0.05
Prospective Data	4 (1584)	0.23	-0.02, 0.45	0.072	0.06

*Note*. Study is used as the the unit of analyses. r = correlation; CI = confidence interval.

^a^ Studies do not add up to 47 due to missing information.

^b^ Anderson LA, Dedrick RF. Development of the Trust in Physician Scale: a measure to assess interpersonal trust in patient-physician relationships. *Psychol Rep* 1990;67:1091–100.

## Discussion

We observed a significant association between trust in the health care professional and health outcome. However, results differed with regard to the outcome dimension, with small and non-significant correlations for objective and observer-rated outcomes and a moderate association with self-rated subjective outcomes. The association between trust and outcome was smaller in high quality studies. Interestingly, the observed association between trust and health appeared to be smaller when binary data were reported and larger, when correlations were reported, and smaller in North America and Asia compared to Europe and Australia.

To the best of our knowledge, this is the first meta-analysis that provides an empirical estimate of the association between trust in the health care professional and health outcome. Our analyses included 34 817 participants from 47 studies in different clinical settings—i.e. disorders and treatment duration varied across as well as within studies and studies were conducted in diverse geographic regions with possibly diverse health care systems.

In order to reduce pragmatic heterogeneity, we included only studies that used a validated trust questionnaire, and we clustered health outcomes into different health outcome dimensions. Furthermore, we checked whether the type of questionnaire moderated study results and confirmed the robustness of the overall finding in a subgroup of 24 studies that all used the same trust questionnaire.

Our meta-analysis has several limitations. First, there are indications that our overall results may be overestimated. We found smaller associations in higher quality studies as well as in larger studies and the significant Egger’s test indicates a lack of small-scaled studies with non-significant correlations (Table 2 and S3 File for funnel plots). However, the large fail-safe N in our analysis indicated a very low risk for a non-significant overall association between trust in the health care professional and health outcome. Second, we were not able to satisfactorily estimate the impact of potential moderators. Here, a reasonable classification of patient characteristics was not possible due to the fact that most samples were mixed with respect to potentially relevant characteristics (e.g. ethnicity or disorder). Also, only six studies reported details on treatment duration. This resulted in a low power of our meta-regression. Statistical heterogeneity, however, was small to moderate in most analyses. This indicates only a small risk for the presence of strong moderators. Finally and most importantly, our analyses do not allow causal interpretations of the observed association between trust in the health care professional and health outcome, since the vast majority of included studies were cross-sectional. However, a subgroup-analysis including only the four prospective studies that assessed trust before the outcome assessment, showed the same moderately sized association between trust and health outcomes as the overall analysis including prospective and cross-sectional studies.

Patients’ trust in the health care professional may best be conceptualized as a contextual factor of treatment effects [10, 11, 34]. Indeed previous work mentions trust in relation to the patient-clinician relationship, which is also embraced by the umbrella-term of contextual factors. Systematic reviews and meta-analyses of RCTs have found small to moderate associations between such factors on health outcomes [13, 34]. Thus, given the risk that the overall association between trust and outcome was overestimated in our meta-analysis, a slightly reduced association in our study could be considered as complementing previous findings.

The differential finding for the type of outcome dimension—i.e. larger associations for the self-rated subjective outcomes and a small or even non-significant association for objective outcomes—has already been described by Beecher in 1955 and has been confirmed for diverse contextual factors ever since [35–38].

A conceptual proximity between trust and subjective outcomes could explain the observed large associations [6, 39]. Indeed, data from a large-scale survey empirically confirms a meaningful association between trust in the health care professional and subjective health [8]. However, since meta-analyses described an enhanced risk of bias with regard to self-rated subjective outcomes, possibly due to inadequate allocation concealment and inappropriate blinding [40, 41], the particularly large associations in our analyses may result—at least partly—from an upward bias.

The non-significant association between trust and objective health outcomes in our meta-analyses may be seen as confirming the previous findings and thus, reflect a *de facto* absence of such an association. However, we observed a significant correlation between trust and self-rated subjective outcomes, which in turn have been associated with objective outcomes [42–44]. Therefore, it could be argued that a possible association between trust and objective outcomes depends on trust-sensitive subjective variables, such as adherence to medication or patient satisfaction with treatment [45, 46]. We could not test the assumption of such a causal chain, however, since the included studies did not stratify their results with regard to possible trust-sensitive subjective variables. Considering that the establishment of interpersonal trust has been described as evolving continuously rather than being a rigid state [47], the cross-sectional study design of most included studies might have hindered the detection of a possible time-delayed impact of trust on objective outcomes.

We found a larger association in studies that reported correlations and a lower association in studies that reported binary data. This pattern was not due to a predominant use of objective outcomes in the studies with binary data and subjective outcomes in the studies with correlational data: Among the studies that reported correlations 33.33% reported objective data, and among the studies that reported binary data 26.67% reported objective data. With regard to the differences in the observed trust-outcome associations in Europe, North America, Australia, and Asia, which are in part also reflected in a recent survey on trust in physicians [48], it is tempting to assume differences in health care systems [49] or in social and cultural factors [50] to account for this finding. However, the available dataset of our meta-analysis did not allow for the testing of these assumptions.

The summarized data indicate that patients report more beneficial health behaviours, higher satisfaction and health-related quality of life, but also better symptom-oriented subjective outcomes when they had higher trust in their health care professional. These findings substantiate the asserted fundamental role of patients’ trust in the context of patient-centered care [9, 18]. It appears tempting to implement the suggested predictors of trust [51, 52] in clinical care as a feasible and possibly cost-effective way to enhance trust and, thus, health outcomes. However, it remains debatable whether such action suffices [18]. First, it must be taken into account that trust and health outcomes may mutually affect each other [6, 43]. Also, trust has been argued to be highly sensitive to more distal, i.e. political, social, and cultural processes [50], which could possibly be reflected in the geographical differences in our analyses. Thus, a sustainable investment should encompass the micro (e.g. patient-provider relationship, quality of health care provision) as well as the macro level (e.g. organisation, financing, and structure of the health care service) [53].

Previous research has proposed different models on how trust could influence health outcomes [45]. For example, Lee and Lin proposed that patients’ trust influences the health outcomes via patient disclosure, the placebo effect, compliance, and the physician’s caring behaviour [54]. Although this model particularly focuses on the association between patients’ trust and health outcomes, it lacks conceptual clarity, for instance with respect to the definition of health outcomes and the influence of more distal factors as well as possible mediators and moderators. Our study highlights the need to differentiate between outcome dimensions. For instance, in accordance with the model proposed by Wampold and Imel, trust in the health care professional might have a positive impact on subjective health (e.g. beneficial health behaviors), which might then, in turn, lead to improvements on objective outcomes [11]. Accordingly, a conceptual clarification of key variables would not only advance the debate on trust, but also allow the deduction of empirically testable hypotheses. The complex interactions between trust and health outcome, including potentially time-delayed effects, reverse causality, as well as the existence of moderators and mediators should further be investigated in prospective studies. Finally, the influence of distal factors (organizational, political, social as well as cultural variables) on the association between trust and outcome needs to be tested and considered in an all-encompassing model.

## Conclusion

Across diverse clinical settings, patients reported to be more satisfied with treatment, to show more beneficial health behaviours, less symptoms and higher quality of life when they had higher trust in their health care professional. But there was no association between trust and observer-rated or trust and objective health outcomes. Although further studies are required to test the direction of the association between trust and health outcome, trust in the health care professional may not only be a deontological constituent of clinical care [55], but it might also be consequential for patients’ treatment satisfaction, health behaviours, symptom severity and quality of life.

## Supporting information

S1 File Search Strategies. (PDF)

S2 File Forest Plots of all conducted meta-analyses and subgroup analyses. (PDF)

S3 File Funnel Plots. (PDF)

S4 File Scatter Plots of meta-regressions. (PDF)

S1 Table Characteristics of Studies Included in the Meta-Analysis. (PDF)
