# Supplementary material for: Trust in the health care professional and health outcome: A meta-analysis
Source: PLoS One. 2017 Feb 7;12(2):e0170988. doi: 10.1371/journal.pone.0170988 (PMC5295692; doi:10.1371/journal.pone.0170988)
Supplement: S1 File — (PDF) [file pone.0170988.s001.pdf]

Supporting File S1. Search Strategies.

Embase (Elsevier) (Harvard library)
1692 Records, 21030621

('trust'/exp AND 'doctor patient relation'/exp) OR (trust* NEAR/2 (patient* OR physician* OR doctor* OR therapist* OR provider* OR nurse*)):ab,ti

PubMed (MEDLINE) (Harvard library)
2475 Records, 20130621

(exp "Trust"/ and exp "Physician-Patient Relations"/) OR (trust* adj2 (patient* OR physician* OR doctor* OR therapist* OR provider* OR nurse*)).tw

CINAHL (EBSCO) (Harvard library)
2218 Records, 20130621

MH ("Professional-Patient Relations+" AND "Trust") OR TI (trust* N2 (patient* OR physician* OR doctor* OR therapist* OR provider* OR nurse*)) OR AB (trust* N2 (patient* OR physician* OR doctor* OR therapist* OR provider* OR nurse*))

PsycINFO (EBSCOhost) (Harvard library)
1509 Records, 20130621

(DE "Trust (Social Behavior)" AND (TI (patient* OR physician* OR doctor* OR therapist* OR provider* OR nurse*) OR AB (patient* OR physician* OR doctor* OR therapist* OR provider* OR nurse*))) OR TI (trust* N2 (patient* OR physician* OR doctor* OR therapist* OR provider* OR nurse*)) OR AB (trust* N2 (patient* OR physician* OR doctor* OR therapist* OR provider* OR nurse*))
