# Supplementary material for: Trust in the health care professional and health outcome: A meta-analysis
Source: PLoS One. 2017 Feb 7;12(2):e0170988. doi: 10.1371/journal.pone.0170988 (PMC5295692; doi:10.1371/journal.pone.0170988)
Supplement: S2 File — (PDF) [file pone.0170988.s002.pdf]

Supporting File S2. Forest Plots.

Analysis including all outcomes

Study name	Subgroup within study	Correlation	Lower limit	Upper limit	p-Value	Relative weight
Altice, 2001	1	0,246	0,094	0,387	0,002	1,98
Anderson*, 1990	Combined	0,326	0,193	0,446	0,000	2,05
Anderson, 1990	1	-0,050	-0,214	0,116	0,557	1,94
Baker, 2003	Combined	0,447	0,417	0,475	0,000	2,38
Balkrishnan, 2003	Combined	0,384	0,347	0,420	0,000	2,37
Becker, 2008	Combined	0,263	0,235	0,290	0,000	2,39
Benkert, 2006	1	0,470	0,332	0,588	0,000	1,95
Biedrzycki, 2011	Combined	0,115	0,034	0,194	0,005	2,28
Blackstock, 2012	1	0,353	0,192	0,496	0,000	1,90
Boothroyd, 2008	1	0,664	0,634	0,692	0,000	2,35
Bosworth, 2004	1	0,142	0,076	0,205	0,000	2,32
Burge, 2009	Combined	0,095	-0,077	0,261	0,280	1,92
Cooper, 2009	Combined	0,102	0,046	0,158	0,000	2,34
Distefano, 1981	1	0,430	0,137	0,654	0,005	1,27
Durant, 2010	1	0,008	-0,036	0,052	0,715	2,37
Farin, 2010	Combined	0,232	0,171	0,291	0,000	2,32
Fernandez, 2012	Combined	-0,035	-0,093	0,023	0,237	2,34
Freburger, 2003	Combined	0,054	0,021	0,086	0,001	2,38
Garcia-Gonzalez, 2009	1	0,430	0,310	0,537	0,000	2,06
Graham, 2013	1	-0,120	-0,265	0,031	0,118	2,01
Gregg,2010	1	0,020	-0,026	0,066	0,390	2,36
Guipponi *, 2009	1	0,448	0,315	0,564	0,000	1,99
Guipponi, 2009	1	0,376	0,196	0,532	0,000	1,80
Hillen, 2013	1	0,620	0,519	0,704	0,000	2,02
Hunfeld,1999	1	0,600	0,259	0,808	0,001	0,93
Kao, 2012	Combined	0,147	0,033	0,258	0,012	2,16
Kerse, 2004	1	0,019	0,006	0,032	0,005	2,40
Lee, 2009	Combined	0,126	0,098	0,153	0,000	2,39
Mainous, 2004	1	0,229	0,036	0,406	0,021	1,80
Mancuso, 2010	Combined	0,254	0,144	0,357	0,000	2,16
Moffet, 2011	Combined	0,015	-0,001	0,030	0,069	2,40
Nguyen, 2009	Combined	0,111	0,071	0,150	0,000	2,37
Ostertag, 2013	Combined	0,165	0,120	0,209	0,000	2,36
Peters, 2007	Combined	0,113	0,018	0,205	0,020	2,23
Piette, 2005	Combined	0,084	0,043	0,126	0,000	2,37
Platonova, 2008	1	0,770	0,734	0,802	0,000	2,27
Rosman,2012	1	0,118	-0,159	0,378	0,406	1,44
Sizer, 2008	1	0,635	0,489	0,746	0,000	1,72
Slean, 2012	1	0,106	0,066	0,145	0,000	2,37
Spain, 2008	Combined	0,022	-0,006	0,051	0,129	2,39
Taha, 2011	1	0,195	0,075	0,309	0,002	2,13
Thüm, 2012	1	0,546	0,383	0,676	0,000	1,75
Trachtenberg, 2005	1	0,170	0,088	0,250	0,000	2,27
Tucker, 2011	Combined	0,286	0,236	0,334	0,000	2,35
Vina, 2012	1	0,013	-0,004	0,031	0,138	2,40
Vissman, 2013	Combined	0,218	0,079	0,349	0,002	2,05
Waters, 2010	1	0,130	0,033	0,224	0,009	2,22
		0,239	0,189	0,288	0,000	

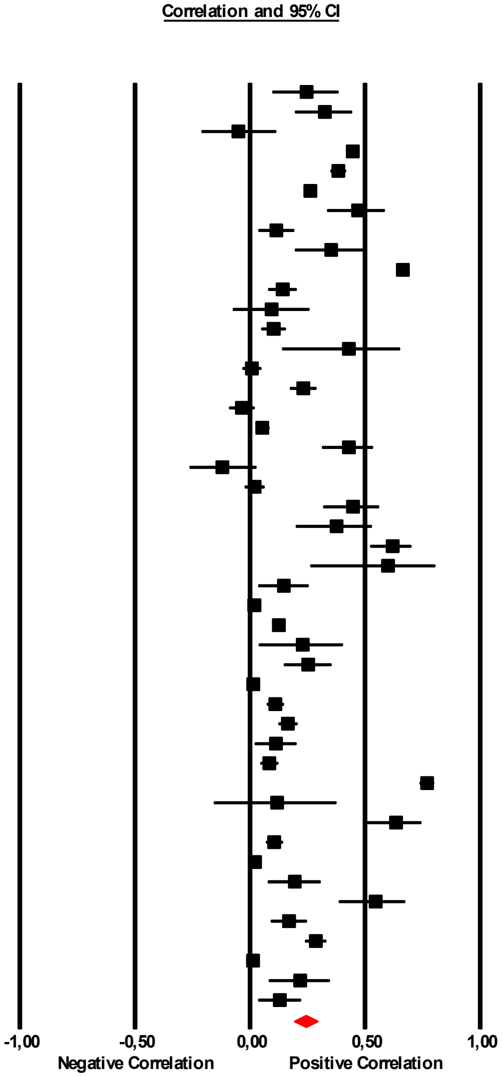

Data set for Forest plot

Study name	Subgroup within study	Correlation	Lower Limit	Upper limit	p-Value
Altice, 2001	1	0,25	0,09	0,39	0,00
Anderson*, 1990	Combined	0,33	0,19	0,45	0,00
Anderson, 1990	1	-0,05	-0,21	0,12	0,56
Baker, 2003	Combined	0,45	0,42	0,48	0,00
Balkrishnan, 2003	Combined	0,38	0,35	0,42	0,00
Becker, 2008	Combined	0,26	0,23	0,29	0,00
Benkert, 2006	1	0,47	0,33	0,59	0,00
Biedrzycki, 2011	Combined	0,12	0,03	0,19	0,01
Blackstock, 2012	1	0,35	0,19	0,50	0,00
Boothroyd, 2008	1	0,66	0,63	0,69	0,00
Bosworth, 2004	1	0,14	0,08	0,21	0,00
Burge, 2009	Combined	0,09	-0,08	0,26	0,28
Cooper, 2009	Combined	0,10	0,05	0,16	0,00
Distefano, 1981	1	0,43	0,14	0,65	0,01
Durant, 2010	1	0,01	-0,04	0,05	0,71
Farin, 2010	Combined	0,23	0,17	0,29	0,00
Fernandez, 2012	Combined	-0,04	-0,09	0,02	0,24
Freburger, 2003	Combined	0,05	0,02	0,09	0,00
Garcia-Gonzalez, 2009	1	0,43	0,31	0,54	0,00
Graham, 2013	1	-0,12	-0,27	0,03	0,12
Gregg, 2010	1	0,02	-0,03	0,07	0,39
Guipponi*, 2009	1	0,45	0,32	0,56	0,00
Guipponi, 2009	1	0,38	0,20	0,53	0,00
Hillen, 2013	1	0,62	0,52	0,70	0,00
Hunfeld, 1999	1	0,60	0,26	0,81	0,00
Kao, 2012	Combined	0,15	0,03	0,26	0,01
Kerse, 2004	1	0,02	0,01	0,03	0,00
Lee, 2009	Combined	0,13	0,10	0,15	0,00
Mainous, 2004	1	0,23	0,04	0,41	0,02
Mancuso, 2010	Combined	0,25	0,14	0,36	0,00
Moffet, 2011	Combined	0,01	0,00	0,03	0,07
Nguyen, 2009	Combined	0,11	0,07	0,15	0,00
Ostertag, 2013	Combined	0,17	0,12	0,21	0,00
Peters, 2007	Combined	0,11	0,02	0,21	0,02
Piette, 2005	Combined	0,08	0,04	0,13	0,00
Platonova, 2008	1	0,77	0,73	0,80	0,00
Rosman, 2012	1	0,12	-0,16	0,38	0,41
Sizer, 2008	1	0,64	0,49	0,75	0,00
Slean, 2012	1	0,11	0,07	0,15	0,00
Spain, 2008	Combined	0,02	-0,01	0,05	0,13

Study name	Subgroup within study	Correlation	Lower limit	Upper limit	p-Value
Thüm, 2012	1	0,55	0,38	0,68	0,00
Trachtenberg, 2005	1	0,17	0,09	0,25	0,00
Tucker, 2011	Combined	0,29	0,24	0,33	0,00
Vina, 2012	1	0,01	0,00	0,03	0,14
Vissman, 2013	Combined	0,22	0,08	0,35	0,00
Waters, 2010	1	0,13	0,03	0,22	0,01
		0,24	0,19	0,29	0,00

Note. *Described as a second independent study population in article, therefore separately included in analysis.

Analysis including objective outcomes

Study name	Subgroup within study	Outcome	Correlation	Lower limit	Upper limit	p-Value	Relative weight
Anderson*, 1990	1,000	2,000	-0,120	-0,319	0,089	0,261	4,12
Anderson, 1990	1,000	2,000	-0,050	-0,214	0,116	0,557	5,24
Balkrishnan, 2003	2,000	2,000	0,100	0,040	0,160	0,001	8,78
Burge, 2009	2,000	2,000	0,073	-0,168	0,306	0,555	3,45
Durant, 2010	1,000	2,000	0,008	-0,035	0,052	0,707	9,24
Fernandez, 2012	Combined	2,000	-0,035	-0,093	0,023	0,237	8,85
Freburger, 2003	Combined	2,000	0,013	-0,030	0,055	0,560	9,27
Graham, 2013	1,000	2,000	-0,120	-0,265	0,031	0,118	5,72
Kao, 2012	Combined	2,000	-0,507	-0,638	-0,348	0,000	4,46
Lee, 2009	Combined	2,000	0,002	-0,035	0,038	0,926	9,40
Mainous, 2004	1,000	2,000	0,229	0,036	0,406	0,021	4,42
Mancuso, 2010	1,000	2,000	0,426	0,252	0,573	0,000	4,43
Ostertag, 2013	3,000	2,000	-0,210	-0,305	-0,111	0,000	7,40
Peters, 2007	Combined	2,000	-0,085	-0,199	0,031	0,151	6,89
Piette, 2005	3,000	2,000	-0,028	-0,103	0,047	0,466	8,31
			-0,023	-0,079	0,033	0,430	

Correlation and 95% CI

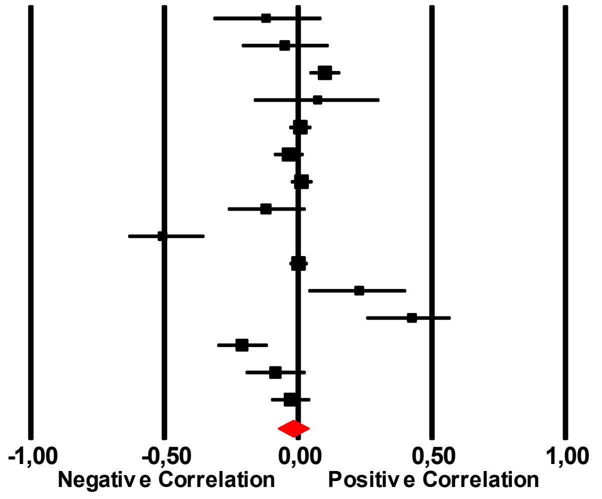

Data set for Forest plot

Study name	Subgroup within study	Outcome	Correlation	Lower limit	Upper limit	p-Value
Anderson*, 1990	1	2	-0,12	-0,32	0,09	0,26
Anderson, 1990	1	2	-0,05	-0,21	0,12	0,56
Balkrishnan, 2003	2	2	0,10	0,04	0,16	0,00
Burge, 2009	2	2	0,07	-0,17	0,31	0,56
Durant, 2010	1	2	0,01	-0,04	0,05	0,71
Fernandez, 2012	Combined	2	-0,04	-0,09	0,02	0,24
Freburger, 2003	Combined	2	0,01	-0,03	0,06	0,56
Graham, 2013	1	2	-0,12	-0,27	0,03	0,12
Kao, 2012	Combined	2	-0,51	-0,64	-0,35	0,00
Lee, 2009	Combined	2	0,00	-0,03	0,04	0,93
Mainous, 2004	1	2	0,23	0,04	0,41	0,02
Mancuso, 2010	1	2	0,43	0,25	0,57	0,00
Ostertag, 2013	3	2	-0,21	-0,30	-0,11	0,00
Peters, 2007	Combined	2	-0,09	-0,20	0,03	0,15
Piette, 2005	3	2	-0,03	-0,10	0,05	0,47
			-0,02	-0,08	0,03	0,43

Note. *Described as a second independent study population in article, therefore separately included in analysis.

Analysis including observer-rated outcomes

	Subgroup within study	Outcome	Correlation	Lower limit	Upper limit	p-Value	Relative weight
Cooper, 2009	Combined	3,000	0,240	0,131	0,344	0,000	48,73
Spain, 2008	2,000	3,000	-0,029	-0,104	0,046	0,448	51,27
			0,104	-0,163	0,357	0,445	

Correlation and 95% CI

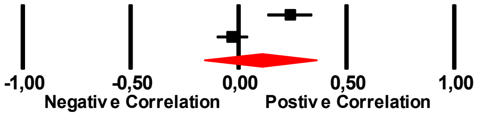

Data set for Forest plot

Study name	Subgroup within study	Outcome	Correlation	Lower limit	Upper limit	p-Value
Cooper, 2009	Combined	3	0,24	0,13	0,34	0,00
Spain, 2008	2	3	-0,03	-0,10	0,05	0,45
			0,10	-0,16	0,36	0,45

Analysis of subjective health outcomes

Study name	Subgroup within study	Outcome	Correlation	Lower limit	Upper limit	p-Value
Altice, 2001	1,000	1,000	0,246	0,094	0,387	0,002
Anderson*, 1990	2,000	1,000	0,620	0,487	0,725	0,000
Baker, 2003	Combined	1,000	0,447	0,417	0,475	0,000
Balkrishnan, 2003	1,000	1,000	0,610	0,570	0,647	0,000
Becker, 2008	Combined	1,000	0,263	0,235	0,290	0,000
Benkert, 2006	1,000	1,000	0,470	0,332	0,588	0,000
Biedrzycki, 2011	Combined	1,000	0,115	0,034	0,194	0,005
Blackstock, 2012	1,000	1,000	0,353	0,192	0,496	0,000
Boothroyd, 2008	1,000	1,000	0,664	0,634	0,692	0,000
Bosworth, 2004	1,000	1,000	0,142	0,076	0,205	0,000
Burge, 2009	1,000	1,000	0,116	-0,126	0,345	0,347
Cooper, 2009	Combined	1,000	0,143	0,078	0,207	0,000
Distefano, 1981	1,000	1,000	0,430	0,137	0,654	0,005
Farin, 2010	Combined	1,000	0,232	0,171	0,291	0,000
Freburger, 2003	Combined	1,000	0,115	0,063	0,166	0,000
Garcia-Gonzalez, 2009	1,000	1,000	0,430	0,310	0,537	0,000
Gregg,2010	1,000	1,000	0,020	-0,026	0,066	0,390
Guipponi *, 2009	1,000	1,000	0,448	0,315	0,564	0,000
Guipponi, 2009	1,000	1,000	0,376	0,196	0,532	0,000
Hillen, 2013	1,000	1,000	0,620	0,519	0,704	0,000
Hunfeld,1999	1,000	1,000	0,600	0,259	0,808	0,001
Kao, 2012	3,000	1,000	0,480	0,363	0,582	0,000
Kerse, 2004	1,000	1,000	0,019	0,006	0,032	0,005
Lee, 2009	Combined	1,000	0,294	0,253	0,335	0,000
Mancuso, 2010	Combined	1,000	0,160	0,022	0,292	0,023
Moffet, 2011	Combined	1,000	0,015	-0,001	0,031	0,058
Nguyen, 2009	Combined	1,000	0,111	0,071	0,150	0,000
Ostertag, 2013	Combined	1,000	0,256	0,208	0,303	0,000
Peters, 2007	3,000	1,000	0,470	0,332	0,588	0,000
Piette, 2005	Combined	1,000	-0,180	-0,236	-0,123	0,000
Platonova, 2008	1,000	1,000	0,770	0,734	0,802	0,000
Rosman,2012	1,000	1,000	0,118	-0,159	0,378	0,406
Sizer, 2008	1,000	1,000	0,635	0,489	0,746	0,000
Slean, 2012	1,000	1,000	0,106	0,066	0,145	0,000
Spain, 2008	1,000	1,000	0,031	0,000	0,062	0,050
Taha, 2011	2,000	1,000	0,195	0,075	0,309	0,002
Thüm, 2012	1,000	1,000	0,546	0,383	0,676	0,000
Trachtenberg, 2005	1,000	1,000	0,170	0,088	0,250	0,000
Tucker, 2011	Combined	1,000	0,286	0,236	0,334	0,000
Vina, 2012	1,000	1,000	0,013	-0,004	0,031	0,138
Vissman, 2013	Combined	1,000	0,218	0,079	0,349	0,002
Waters, 2010	1,000	1,000	-0,130	-0,224	-0,033	0,009
			0,296	0,236	0,354	0,000

Correlation and 95% CI

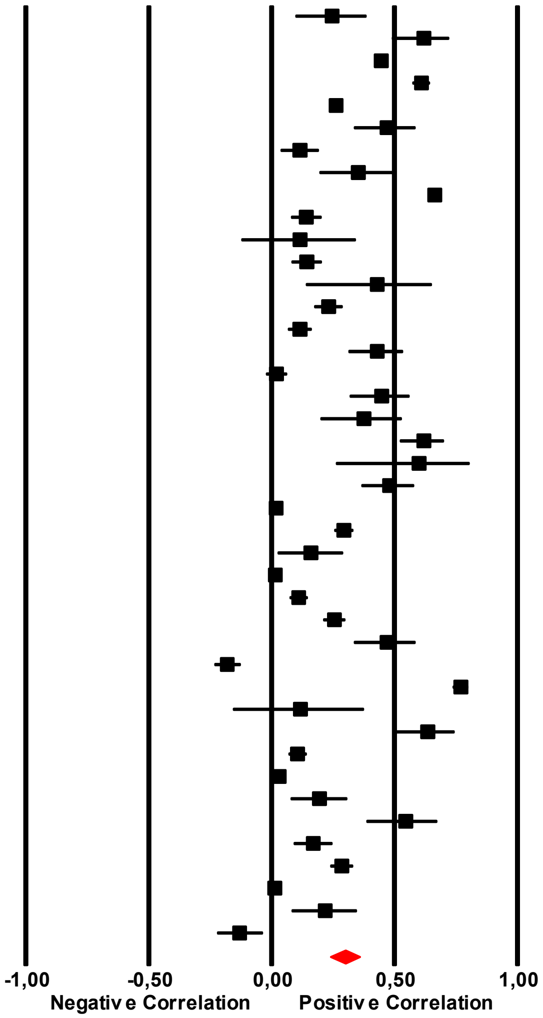

Data set for Forest plot

Study name	Subgroup within study	Outcome	Correlation	Lower limit	Upper limit	p-Value
Altice, 2001	1	1	0,25	0,09	0,39	0,00
Anderson*, 1990	2	1	0,62	0,49	0,73	0,00
Baker, 2003	Combined	1	0,45	0,42	0,48	0,00
Balkrishnan, 2003	1	1	0,61	0,57	0,65	0,00
Becker, 2008	Combined	1	0,26	0,23	0,29	0,00
Benkert, 2006	1	1	0,47	0,33	0,59	0,00
Biedrzycki, 2011	Combined	1	0,12	0,03	0,19	0,01
Blackstock, 2012	1	1	0,35	0,19	0,50	0,00
Boothroyd, 2008	1	1	0,66	0,63	0,69	0,00
Bosworth, 2004	1	1	0,14	0,08	0,21	0,00
Burge, 2009	1	1	0,12	-0,13	0,34	0,35
Cooper, 2009	Combined	1	0,14	0,08	0,21	0,00
Distefano, 1981	1	1	0,43	0,14	0,65	0,01
Farin, 2010	Combined	1	0,23	0,17	0,29	0,00
Freburger, 2003	Combined	1	0,12	0,06	0,17	0,00
Garcia-Gonzalez, 2009	1	1	0,43	0,31	0,54	0,00
Gregg, 2010	1	1	0,02	-0,03	0,07	0,39
Guipponi *, 2009	1	1	0,45	0,32	0,56	0,00
Guipponi, 2009	1	1	0,38	0,20	0,53	0,00
Hillen, 2013	1	1	0,62	0,52	0,70	0,00
Hunfeld, 1999	1	1	0,60	0,26	0,81	0,00
Kao, 2012	3	1	0,48	0,36	0,58	0,00
Kerse, 2004	1	1	0,02	0,01	0,03	0,00
Lee, 2009	Combined	1	0,29	0,25	0,33	0,00
Mancuso, 2010	Combined	1	0,16	0,02	0,29	0,02
Moffet, 2011	Combined	1	0,02	0,00	0,03	0,06
Nguyen, 2009	Combined	1	0,11	0,07	0,15	0,00
Ostertag, 2013	Combined	1	0,26	0,21	0,30	0,00
Peters, 2007	3	1	0,47	0,33	0,59	0,00
Piette, 2005	Combined	1	-0,18	-0,24	-0,12	0,00
Platonova, 2008	1	1	0,77	0,73	0,80	0,00
Rosman, 2012	1	1	0,12	-0,16	0,38	0,41
Sizer, 2008	1	1	0,64	0,49	0,75	0,00
Slean, 2012	1	1	0,11	0,07	0,15	0,00

Spain, 2008	1	1	0,03	0,00	0,06	0,05
Taha, 2011	2	1	0,20	0,08	0,31	0,00
Thüm, 2012	1	1	0,55	0,38	0,68	0,00
Trachtenberg, 2005	1	1	0,17	0,09	0,25	0,00
Tucker, 2011	Combined	1	0,29	0,24	0,33	0,00
Vina, 2012	1	1	0,01	0,00	0,03	0,14
Vissman, 2013	Combined	1	0,22	0,08	0,35	0,00
Waters, 2010	1	1	-0,13	-0,22	-0,03	0,01
			0,30	0,24	0,35	0,00

Analysis including health behavior as outcome

Study name	Subgroup within study	Outcome	Correlation	Lower limit	Upper limit	p-Value
Altice, 2001	1,000	1,000	0,246	0,094	0,387	0,002
Becker, 2008	Combined	1,000	0,263	0,235	0,290	0,000
Blackstock, 2012	1,000	1,000	0,353	0,192	0,496	0,000
Bosworth, 2004	1,000	1,000	0,142	0,076	0,205	0,000
Durant, 2010	1,000	1,000	0,008	-0,035	0,052	0,707
Farin, 2010	Combined	1,000	0,244	0,169	0,316	0,000
Freburger, 2003	4,000	1,000	0,100	0,027	0,172	0,008
Gregg,2010	1,000	1,000	0,020	-0,026	0,066	0,390
Kao, 2012	3,000	1,000	0,480	0,363	0,582	0,000
Kerse, 2004	1,000	1,000	0,019	0,006	0,032	0,005
Lee, 2009	Combined	1,000	0,302	0,243	0,358	0,000
Mancuso, 2010	2,000	1,000	0,108	-0,088	0,296	0,281
Moffet, 2011	Combined	1,000	0,015	-0,001	0,031	0,058
Nguyen, 2009	Combined	1,000	0,111	0,071	0,150	0,000
Ostertag, 2013	Combined	1,000	0,251	0,183	0,317	0,000
Piette, 2005	1,000	1,000	-0,174	-0,264	-0,080	0,000
Rosman,2012	1,000	1,000	0,118	-0,159	0,378	0,406
Trachtenberg, 2005	1,000	1,000	0,170	0,088	0,250	0,000
Tucker, 2011	Combined	1,000	0,158	0,094	0,221	0,000
Vina, 2012	1,000	1,000	0,013	-0,004	0,031	0,138
Vissman, 2013	Combined	1,000	0,218	0,079	0,349	0,002
			0,143	0,098	0,187	0,000

Correlation and 95% CI

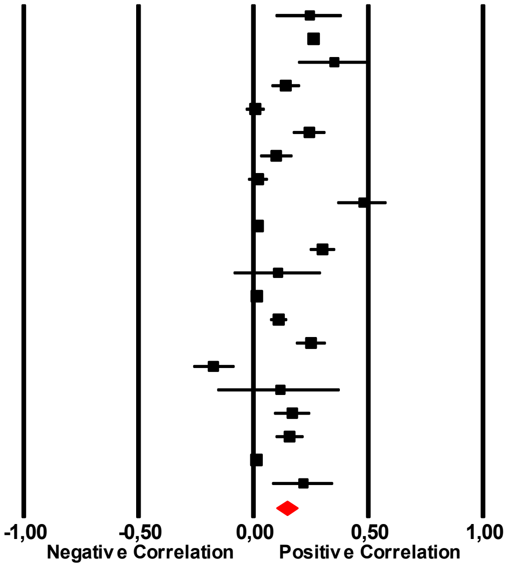

Data set for Forest plot

	Subgroup within study	Outcome	Correlation	Lower limit	Upper limit	p-Value
Altice, 2001	1	1	0,25	0,09	0,39	0,00
Becker, 2008	Combined	1	0,26	0,23	0,29	0,00
Blackstock, 2012	1	1	0,35	0,19	0,50	0,00
Bosworth, 2004	1	1	0,14	0,08	0,21	0,00
Durant, 2010	1	1	0,01	-0,04	0,05	0,71
Farin, 2010	Combined	1	0,24	0,17	0,32	0,00
Freburger, 2003	4	1	0,10	0,03	0,17	0,01
Gregg, 2010	1	1	0,02	-0,03	0,07	0,39
Kao, 2012	3	1	0,48	0,36	0,58	0,00
Kerse, 2004	1	1	0,02	0,01	0,03	0,00
Lee, 2009	Combined	1	0,30	0,24	0,36	0,00
Mancuso, 2010	2	1	0,11	-0,09	0,30	0,28
Moffet, 2011	Combined	1	0,02	0,00	0,03	0,06
Nguyen, 2009	Combined	1	0,11	0,07	0,15	0,00
Ostertag, 2013	Combined	1	0,25	0,18	0,32	0,00
Piette, 2005	1	1	-0,17	-0,26	-0,08	0,00
Rosman, 2012	1	1	0,12	-0,16	0,38	0,41
Trachtenberg, 2005	1	1	0,17	0,09	0,25	0,00
Tucker, 2011	Combined	1	0,16	0,09	0,22	0,00
Vina, 2012	1	1	0,01	0,00	0,03	0,14
Vissman, 2013	Combined	1	0,22	0,08	0,35	0,00
			0,14	0,10	0,19	0,00

Analysis including health-related subjective experience as outcome

Study name	Subgroup within study	Outcome	Correlation	Lower limit	Upper limit	p-Value	Relative weight
Anderson*, 1990	2,000	2,000	0,620	0,487	0,725	0,000	3,32
Baker, 2003	Combined	2,000	0,447	0,417	0,475	0,000	3,65
Balkrishnan, 2003	1,000	2,000	0,610	0,570	0,647	0,000	3,63
Benkert, 2006	1,000	2,000	0,470	0,332	0,588	0,000	3,41
Biedrzycki, 2011	Combined	2,000	0,115	0,034	0,194	0,005	3,60
Boothroyd, 2008	1,000	2,000	0,664	0,634	0,692	0,000	3,64
Burge, 2009	Combined	2,000	0,095	-0,077	0,261	0,280	3,39
Cooper, 2009	Combined	2,000	0,055	-0,010	0,121	0,099	3,62
Distefano, 1981	1,000	2,000	0,430	0,137	0,654	0,005	2,84
Farin, 2010	1,000	2,000	0,208	0,100	0,311	0,000	3,55
Freburger, 2003	5,000	2,000	0,130	0,057	0,202	0,000	3,61
Garcia-Gonzalez, 2009	1,000	2,000	0,430	0,310	0,537	0,000	3,48
Guipponi *, 2009	1,000	2,000	0,448	0,315	0,564	0,000	3,43
Guipponi, 2009	1,000	2,000	0,376	0,196	0,532	0,000	3,31
Hillen, 2013	1,000	2,000	0,620	0,519	0,704	0,000	3,45
Hunfeld,1999	1,000	2,000	0,600	0,259	0,808	0,001	2,43
Lee, 2009	Combined	2,000	0,287	0,227	0,344	0,000	3,62
Mancuso, 2010	3,000	2,000	0,211	0,017	0,389	0,033	3,31
Ostertag, 2013	Combined	2,000	0,260	0,192	0,326	0,000	3,61
Peters, 2007	3,000	2,000	0,470	0,332	0,588	0,000	3,41
Piette, 2005	2,000	2,000	-0,184	-0,254	-0,112	0,000	3,61
Platonova, 2008	1,000	2,000	0,770	0,734	0,802	0,000	3,60
Sizer, 2008	1,000	2,000	0,635	0,489	0,746	0,000	3,25
Slean, 2012	1,000	2,000	0,106	0,066	0,145	0,000	3,65
Spain, 2008	1,000	2,000	0,031	0,000	0,062	0,050	3,65
Taha, 2011	2,000	2,000	0,195	0,075	0,309	0,002	3,52
Thüm, 2012	1,000	2,000	0,546	0,383	0,676	0,000	3,27
Tucker, 2011	Combined	2,000	0,511	0,439	0,576	0,000	3,58
Waters, 2010	1,000	2,000	0,130	0,033	0,224	0,009	3,57
			0,373	0,270	0,467	0,000	

Correlation and 95% CI

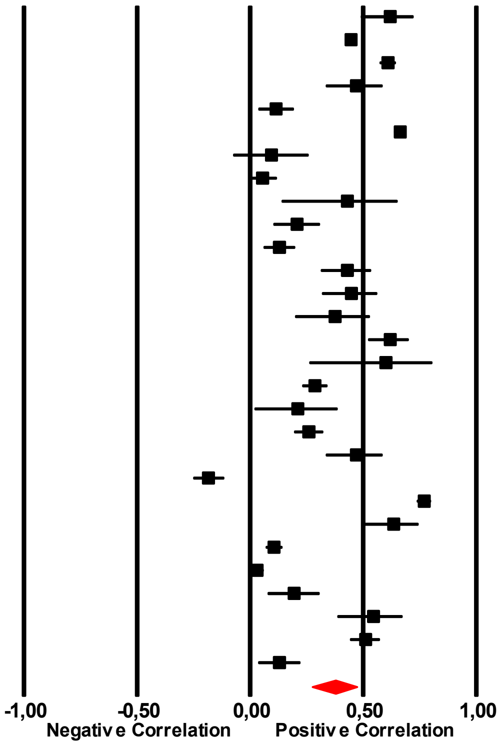

Data set for Forest plot

Study name	Subgroup within study	Outcome	Correlation	Lower limit	Upper limit	p-Value
Anderson*, 1990	2	2	0,62	0,49	0,73	0,00
Baker, 2003	Combined	2	0,45	0,42	0,48	0,00
Balkrishnan, 2003	1	2	0,61	0,57	0,65	0,00
Benkert, 2006	1	2	0,47	0,33	0,59	0,00
Biedrzycki, 2011	Combined	2	0,12	0,03	0,19	0,01
Boothroyd, 2008	1	2	0,66	0,63	0,69	0,00
Burge, 2009	Combined	2	0,09	-0,08	0,26	0,28
Cooper, 2009	Combined	2	0,06	-0,01	0,12	0,10
Distefano, 1981	1	2	0,43	0,14	0,65	0,01
Farin, 2010	1	2	0,21	0,10	0,31	0,00
Freburger, 2003	5	2	0,13	0,06	0,20	0,00
Garcia-Gonzalez, 2009	1	2	0,43	0,31	0,54	0,00
Guipponi*, 2009	1	2	0,45	0,32	0,56	0,00
Guipponi, 2009	1	2	0,38	0,20	0,53	0,00
Hillen, 2013	1	2	0,62	0,52	0,70	0,00
Hunfeld, 1999	1	2	0,60	0,26	0,81	0,00
Lee, 2009	Combined	2	0,29	0,23	0,34	0,00
Mancuso, 2010	3	2	0,21	0,02	0,39	0,03
Ostertag, 2013	Combined	2	0,26	0,19	0,33	0,00
Peters, 2007	3	2	0,47	0,33	0,59	0,00
Piette, 2005	2	2	-0,18	-0,25	-0,11	0,00
Platonova, 2008	1	2	0,77	0,73	0,80	0,00
Sizer, 2008	1	2	0,64	0,49	0,75	0,00
Slean, 2012	1	2	0,11	0,07	0,15	0,00
Spain, 2008	1	2	0,03	0,00	0,06	0,05
Taha, 2011	2	2	0,20	0,08	0,31	0,00
Thüm, 2012	1	2	0,55	0,38	0,68	0,00
Tucker, 2011	Combined	2	0,51	0,44	0,58	0,00
Waters, 2010	1	2	0,13	0,03	0,22	0,01
			0,37	0,27	0,47	0,00

Note. *Described as a second independent study population in article, therefore separately included in analysis.

Analysis of patient satisfaction as outcome

Study name	Subgroup within study	Outcome	Correlation	Lower limit	Upper limit	p-Value	Relative weight
Anderson*, 1990	2,000	2,000	0,620	0,487	0,725	0,000	6,42
Baker, 2003	Combined	2,000	0,447	0,417	0,475	0,000	7,88
Balkrishnan, 2003	1,000	2,000	0,610	0,570	0,647	0,000	7,76
Benkert, 2006	1,000	2,000	0,470	0,332	0,588	0,000	6,77
Boothroyd, 2008	1,000	2,000	0,664	0,634	0,692	0,000	7,81
Distefano, 1981	1,000	2,000	0,430	0,137	0,654	0,005	4,78
Garcia-Gonzalez, 2009	1,000	2,000	0,430	0,310	0,537	0,000	7,06
Guipponi *, 2009	1,000	2,000	0,448	0,315	0,564	0,000	6,88
Guipponi, 2009	1,000	2,000	0,376	0,196	0,532	0,000	6,37
Hillen, 2013	1,000	2,000	0,620	0,519	0,704	0,000	6,95
Hunfeld,1999	1,000	2,000	0,600	0,259	0,808	0,001	3,66
Peters, 2007	3,000	2,000	0,470	0,332	0,588	0,000	6,77
Platonova, 2008	1,000	2,000	0,770	0,734	0,802	0,000	7,60
Sizer, 2008	1,000	2,000	0,635	0,489	0,746	0,000	6,13
Tucker, 2011	2,000	2,000	0,747	0,684	0,799	0,000	7,16
			0,573	0,493	0,643	0,000	

Correlation and 95% CI

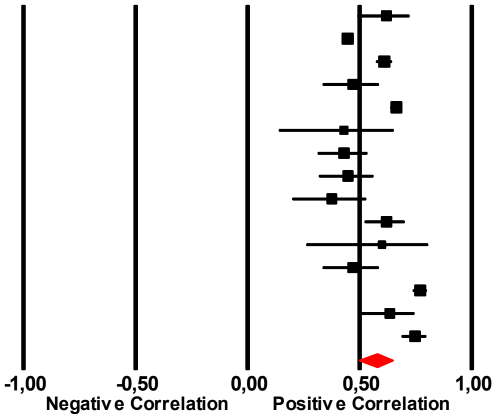

Data set for Forest plot

	Subgroup within study	Outcome	Correlation	Lower limit	Upper limit	p-Value
Altice, 2001	1	1	0,25	0,09	0,39	0,00
Becker, 2008	Combined	1	0,26	0,23	0,29	0,00
Blackstock, 2012	1	1	0,35	0,19	0,50	0,00
Bosworth, 2004	1	1	0,14	0,08	0,21	0,00
Durant, 2010	1	1	0,01	-0,04	0,05	0,71
Farin, 2010	Combined	1	0,24	0,17	0,32	0,00
Freburger, 2003	4	1	0,10	0,03	0,17	0,01
Gregg, 2010	1	1	0,02	-0,03	0,07	0,39
Kao, 2012	3	1	0,48	0,36	0,58	0,00
Kerse, 2004	1	1	0,02	0,01	0,03	0,00
Lee, 2009	Combined	1	0,30	0,24	0,36	0,00
Mancuso, 2010	2	1	0,11	-0,09	0,30	0,28
Moffet, 2011	Combined	1	0,02	0,00	0,03	0,06
Nguyen, 2009	Combined	1	0,11	0,07	0,15	0,00
Ostertag, 2013	Combined	1	0,25	0,18	0,32	0,00
Piette, 2005	1	1	-0,17	-0,26	-0,08	0,00
Rosman, 2012	1	1	0,12	-0,16	0,38	0,41
Trachtenberg, 2005	1	1	0,17	0,09	0,25	0,00
Tucker, 2011	Combined	1	0,16	0,09	0,22	0,00
Vina, 2012	1	1	0,01	0,00	0,03	0,14
Vissman, 2013	Combined	1	0,22	0,08	0,35	0,00
			0,14	0,10	0,19	0,00

Note. *Described as a second independent study population in article, therefore separately included in analysis.

Analysis of health related quality of life as outcome

Study name	Subgroup within study	Outcome	Correlation	Lower limit	Upper limit	p-Value	Relative weight
Biedrzycki, 2011	3,000	1,000	0,228	0,091	0,356	0,001	8,49
Cooper, 2009	Combined	1,000	0,195	0,117	0,271	0,000	25,90
Farin, 2010	1,000	1,000	0,208	0,100	0,311	0,000	13,82
Freburger, 2003	5,000	1,000	0,130	0,057	0,202	0,000	31,06
Lee, 2009	Combined	1,000	0,187	0,099	0,272	0,000	20,73
			0,178	0,138	0,217	0,000	

Correlation and 95% CI

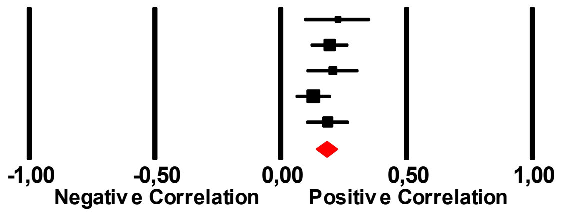

Data set for Forest plot

Study name	Subgroup within study	Outcome	Correlation	Lower limit	Upper limit	p-Value
Biedrzycki, 2011	3	1	0,23	0,09	0,36	0,00
Cooper, 2009	Combined	1	0,20	0,12	0,27	0,00
Farin, 2010	1	1	0,21	0,10	0,31	0,00
Freburger, 2003	5	1	0,13	0,06	0,20	0,00
Lee, 2009	Combined	1	0,19	0,10	0,27	0,00
			0,18	0,14	0,22	0,00

Analysis including symptom related outcomes

Study name	Subgroup within study	Outcome	Statistics for each study: Correlation	Lower limit	Upper limit	p-Value	Relative weight
Biedrzycki, 2011	Combined	1,000	0,057	-0,042	0,156	0,259	7,98
Burge, 2009	Combined	1,000	0,095	-0,077	0,261	0,280	6,68
Cooper, 2009	Combined	1,000	-0,225	-0,330	-0,115	0,000	7,75
Lee, 2009	10,000	1,000	0,380	0,301	0,454	0,000	8,13
Mancuso, 2010	3,000	1,000	0,211	0,017	0,389	0,033	6,20
Ostertag, 2013	Combined	1,000	0,260	0,192	0,326	0,000	8,38
Piette, 2005	2,000	1,000	-0,184	-0,254	-0,112	0,000	8,36
Slean, 2012	1,000	1,000	0,106	0,066	0,145	0,000	8,70
Spain, 2008	1,000	1,000	0,031	0,000	0,062	0,050	8,76
Taha, 2011	2,000	1,000	0,195	0,075	0,309	0,002	7,60
Thüm, 2012	1,000	1,000	0,546	0,383	0,676	0,000	5,97
Tucker, 2011	3,000	1,000	0,160	0,031	0,284	0,015	7,46
Waters, 2010	1,000	1,000	0,130	0,033	0,224	0,009	8,02
			0,131	0,042	0,218	0,004	

Correlation and 95% CI

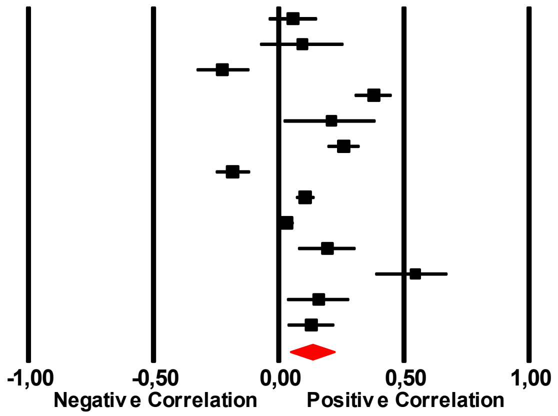

Data set for Forest plot

Study name	Subgroup within study	Outcome	Correlation	Lower limit	Upper limit	p-Value
Biedrzycki, 2011	Combined	1	0,06	-0,04	0,16	0,26
Burge, 2009	Combined	1	0,09	-0,08	0,26	0,28
Cooper, 2009	Combined	1	-0,23	-0,33	-0,11	0,00
Lee, 2009	10	1	0,38	0,30	0,45	0,00
Mancuso, 2010	3	1	0,21	0,02	0,39	0,03
Ostertag, 2013	Combined	1	0,26	0,19	0,33	0,00
Piette, 2005	2	1	-0,18	-0,25	-0,11	0,00
Slean, 2012	1	1	0,11	0,07	0,15	0,00
Spain, 2008	1	1	0,03	0,00	0,06	0,05
Taha, 2011	2	1	0,20	0,08	0,31	0,00
Thüm, 2012	1	1	0,55	0,38	0,68	0,00
Tucker, 2011	3	1	0,16	0,03	0,28	0,02
Waters, 2010	1	1	0,13	0,03	0,22	0,01
			0,13	0,04	0,22	0,00

Analysis including correlational data

Study name	Subgroup within study	Correlation	Lower limit	Upper limit	p-Value	Relative weight
Anderson*, 1990	Combined	0,326	0,193	0,446	0,000	0,64
Anderson, 1990	1	-0,050	-0,214	0,116	0,557	0,46
Baker, 2003	Combined	0,447	0,417	0,475	0,000	9,80
Balkrishnan, 2003	Combined	0,384	0,347	0,420	0,000	7,00
Becker, 2008	Combined	0,263	0,235	0,290	0,000	14,44
Benkert, 2006	1	0,470	0,332	0,588	0,000	0,48
Biedrzycki, 2011	Combined	0,115	0,034	0,194	0,005	1,96
Boothroyd, 2008	1	0,664	0,634	0,692	0,000	4,71
Burge, 2009	Combined	0,095	-0,077	0,261	0,280	0,44
Cooper, 2009	Combined	0,102	0,046	0,158	0,000	3,98
Distefano, 1981	1	0,430	0,137	0,654	0,005	0,12
Farin, 2010	Combined	0,232	0,171	0,291	0,000	3,19
Freburger, 2003	Combined	0,054	0,021	0,086	0,001	11,93
Garcia-Gonzalez, 2009	1	0,430	0,310	0,537	0,000	0,66
Graham, 2013	1	-0,120	-0,265	0,031	0,118	0,56
Guipponi *, 2009	1	0,448	0,315	0,564	0,000	0,53
Guipponi, 2009	1	0,376	0,196	0,532	0,000	0,33
Hillen, 2013	1	0,620	0,519	0,704	0,000	0,58
Hunfeld,1999	1	0,600	0,259	0,808	0,001	0,07
Kao, 2012	Combined	0,147	0,033	0,258	0,012	0,97
Lee, 2009	Combined	0,126	0,098	0,153	0,000	16,02
Mancuso, 2010	Combined	0,254	0,144	0,357	0,000	1,00
Nguyen, 2009	1	0,300	0,179	0,412	0,000	0,78
Ostertag, 2013	Combined	0,165	0,120	0,209	0,000	6,23
Peters, 2007	Combined	0,113	0,018	0,205	0,020	1,43
Platonova, 2008	1	0,770	0,734	0,802	0,000	1,85
Sizer, 2008	1	0,635	0,489	0,746	0,000	0,28
Taha, 2011	1	0,195	0,075	0,309	0,002	0,86
Thüm, 2012	1	0,546	0,383	0,676	0,000	0,30
Trachtenberg, 2005	1	0,170	0,088	0,250	0,000	1,85
Tucker, 2011	Combined	0,286	0,236	0,334	0,000	4,56
Vissman, 2013	Combined	0,218	0,079	0,349	0,002	0,64
Waters, 2010	1	0,130	0,033	0,224	0,009	1,36
		0,265	0,254	0,275	0,000	

Correlation and 95% CI

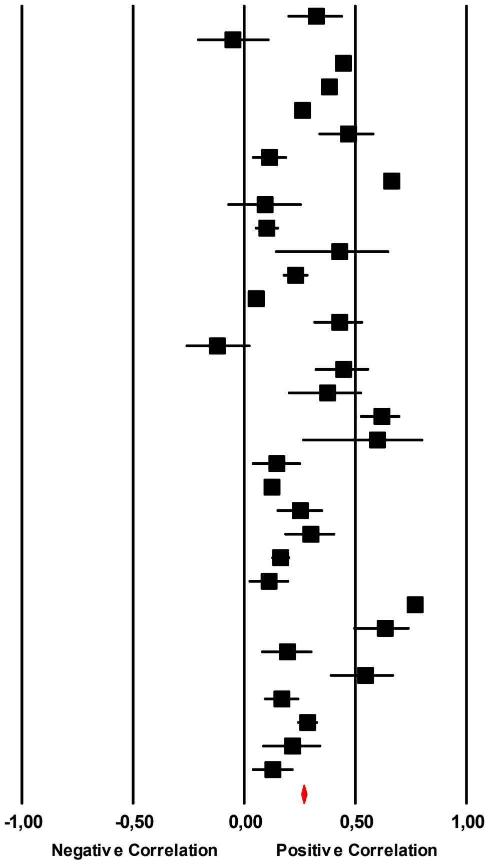

Data set for Forest plot

Study name	Subgroup within study	Correlation	Lower limit	Upper limit	p-Value
Anderson*, 1990	Combined	0,33	0,19	0,45	0,00
Anderson, 1990	1,00	-0,05	-0,21	0,12	0,56
Baker, 2003	Combined	0,45	0,42	0,48	0,00
Balkrishnan, 2003	Combined	0,38	0,35	0,42	0,00
Becker, 2008	Combined	0,26	0,23	0,29	0,00
Benkert, 2006	1,00	0,47	0,33	0,59	0,00
Biedrzycki, 2011	Combined	0,12	0,03	0,19	0,01
Boothroyd, 2008	1,00	0,66	0,63	0,69	0,00
Burge, 2009	Combined	0,09	-0,08	0,26	0,28
Cooper, 2009	Combined	0,10	0,05	0,16	0,00
Distefano, 1981	1,00	0,43	0,14	0,65	0,01
Farin, 2010	Combined	0,23	0,17	0,29	0,00
Freburger, 2003	Combined	0,05	0,02	0,09	0,00
Garcia-Gonzalez, 2009	1,00	0,43	0,31	0,54	0,00
Graham, 2013	1,00	-0,12	-0,27	0,03	0,12
Guipponi *, 2009	1,00	0,45	0,32	0,56	0,00
Guipponi, 2009	1,00	0,38	0,20	0,53	0,00
Hillen, 2013	1,00	0,62	0,52	0,70	0,00
Hunfeld,1999	1,00	0,60	0,26	0,81	0,00
Kao, 2012	Combined	0,15	0,03	0,26	0,01
Lee, 2009	Combined	0,13	0,10	0,15	0,00
Mancuso, 2010	Combined	0,25	0,14	0,36	0,00
Nguyen, 2009	1,00	0,30	0,18	0,41	0,00
Ostertag, 2013	Combined	0,17	0,12	0,21	0,00
Peters, 2007	Combined	0,11	0,02	0,21	0,02
Platonova, 2008	1,00	0,77	0,73	0,80	0,00
Sizer, 2008	1,00	0,64	0,49	0,75	0,00
Taha, 2011	1,00	0,20	0,08	0,31	0,00
Thüm, 2012	1,00	0,55	0,38	0,68	0,00
Trachtenberg, 2005	1,00	0,17	0,09	0,25	0,00
Tucker, 2011	Combined	0,29	0,24	0,33	0,00
Vissman, 2013	Combined	0,22	0,08	0,35	0,00
Waters, 2010	1,00	0,13	0,03	0,22	0,01
		0,31	0,24	0,38	0,00

Note. *Described as a second independent study population in article, therefore separately included in analysis.

Analysis including binary data

Studyname	Subgroup within study	Correlation	Lower limit	Upper limit	p-Value	Relative weight
Altice, 2001	1	0,246	0,094	0,387	0,002	1,82
Blackstock, 2012	1	0,353	0,192	0,496	0,000	1,52
Bosworth, 2004	1	0,142	0,076	0,205	0,000	5,88
Durant, 2010	1	0,008	-0,036	0,052	0,715	8,03
Fernandez, 2012	Combined	-0,035	-0,093	0,023	0,237	6,56
Gregg,2010	1	0,020	-0,026	0,066	0,390	7,82
Kerse, 2004	1	0,019	0,006	0,032	0,005	10,87
Mainous, 2004	1	0,229	0,036	0,406	0,021	1,22
Moffet, 2011	Combined	0,015	-0,001	0,030	0,069	10,70
Nguyen, 2009	Combined	0,089	0,048	0,131	0,000	8,20
Piette, 2005	Combined	0,084	0,043	0,126	0,000	8,21
Rosman,2012	1	0,118	-0,159	0,378	0,406	0,65
Slean, 2012	1	0,106	0,066	0,145	0,000	8,42
Spain, 2008	Combined	0,022	-0,006	0,051	0,129	9,58
Vina, 2012	1	0,013	-0,004	0,031	0,138	10,55
		0,052	0,029	0,075	0,000	

Correlation and 95% CI

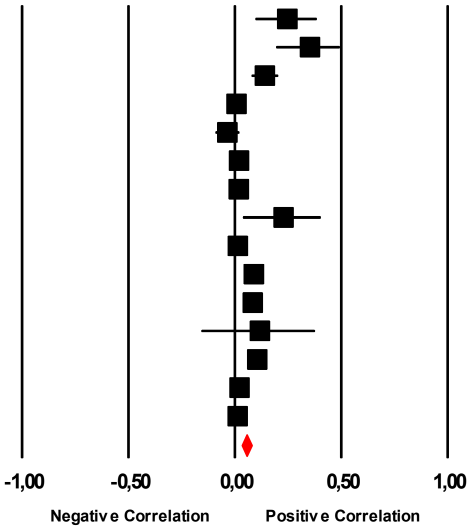

Data set for Forest plot

Study name	Subgroup within study	Correlation	Lower limit	Upper limit	p-Value
Altice, 2001	1	0,25	0,09	0,39	0,00
Blackstock, 2012	1	0,35	0,19	0,50	0,00
Bosworth, 2004	1	0,14	0,08	0,21	0,00
Durant, 2010	1	0,01	-0,04	0,05	0,71
Fernandez, 2012	Combined	-0,04	-0,09	0,02	0,24
Gregg,2010	1	0,02	-0,03	0,07	0,39
Kerse, 2004	1	0,02	0,01	0,03	0,00
Mainous, 2004	1	0,23	0,04	0,41	0,02
Moffet, 2011	Combined	0,01	0,00	0,03	0,07
Nguyen, 2009	Combined	0,09	0,05	0,13	0,00
Piette, 2005	Combined	0,08	0,04	0,13	0,00
Rosman,2012	1	0,12	-0,16	0,38	0,41
Slean, 2012	1	0,11	0,07	0,15	0,00
Spain, 2008	Combined	0,02	-0,01	0,05	0,13
Vina, 2012	1	0,01	0,00	0,03	0,14
		0,05	0,03	0,08	0,00

Analysis including prospective data

Studyname	Subgroup within study	Correlation	Lower limit	Upper limit	p-Value	Relative weight
Anderson*, 1990	Combined	0,326	0,193	0,446	0,000	25,87
Anderson, 1990	1	-0,050	-0,214	0,116	0,557	25,11
Baker, 2003	Combined	0,447	0,417	0,475	0,000	27,97
Rosman,2012	1	0,118	-0,159	0,378	0,406	21,05
		0,230	-0,021	0,454	0,072	

Correlation and 95% CI

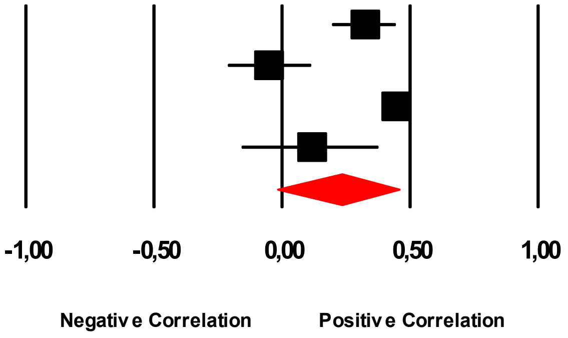

Data set for Forest plot

Study name	Subgroup within study	Correlation	Lower limit	Upper limit	p-Value
Anderson*, 1990	Combined	0,33	0,19	0,45	0,00
Anderson, 1990	1,00	-0,05	-0,21	0,12	0,56
Baker, 2003	Combined	0,45	0,42	0,48	0,00
Rosman,2012	1,00	0,12	-0,16	0,38	0,41
		0,23	-0,02	0,45	0,07

Note. *Described as a second independent study population in article, therefore separately included in analysis.
