# Supplementary material for: Trust in the health care professional and health outcome: A meta-analysis
Source: PLoS One. 2017 Feb 7;12(2):e0170988. doi: 10.1371/journal.pone.0170988 (PMC5295692; doi:10.1371/journal.pone.0170988)

Supporting File S3. Funnel Plots.

1. **Funnel plot: Analysis including all outcomes**

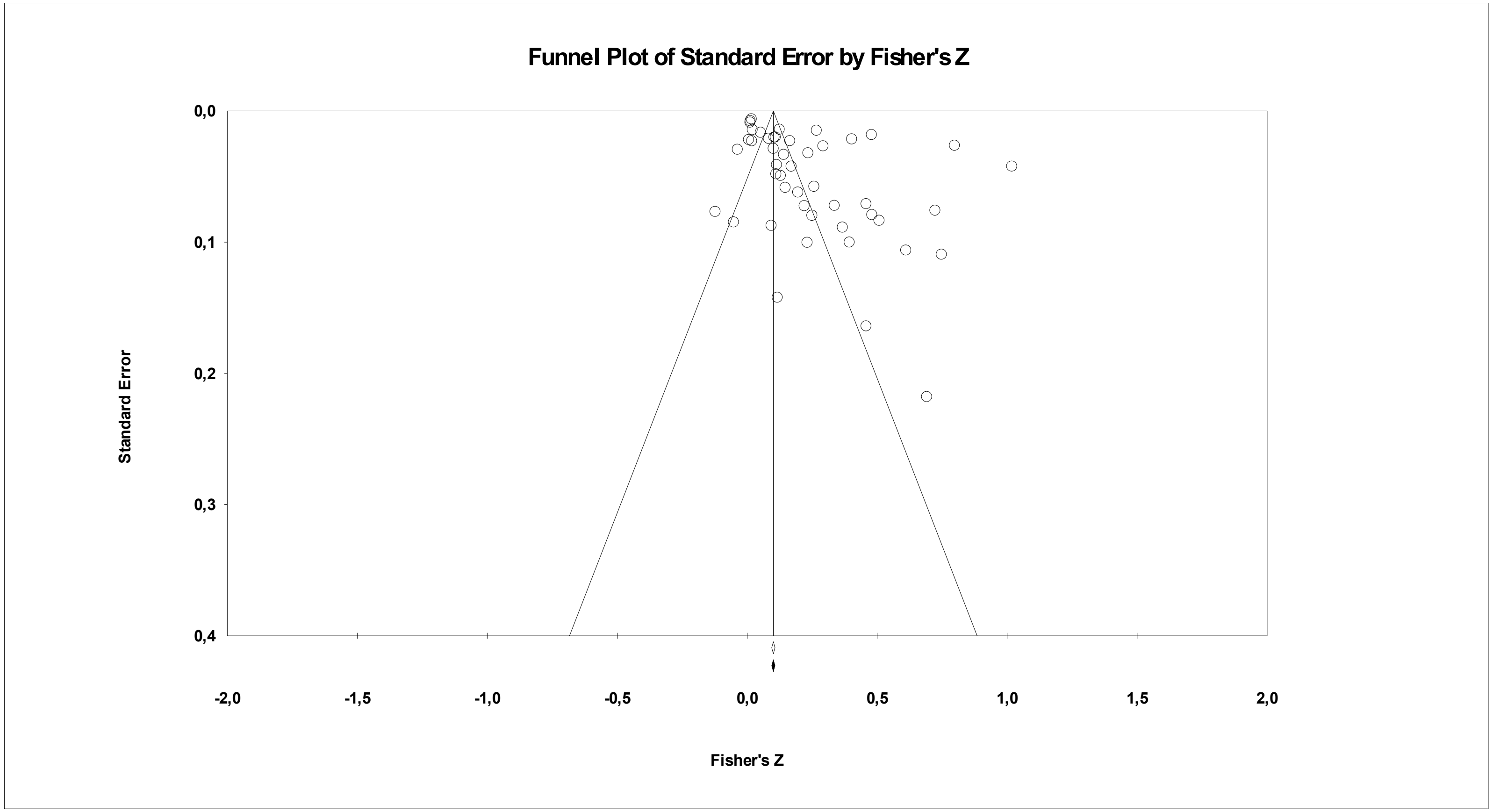

2. Funnel plot: Analysis including objective health outcomes

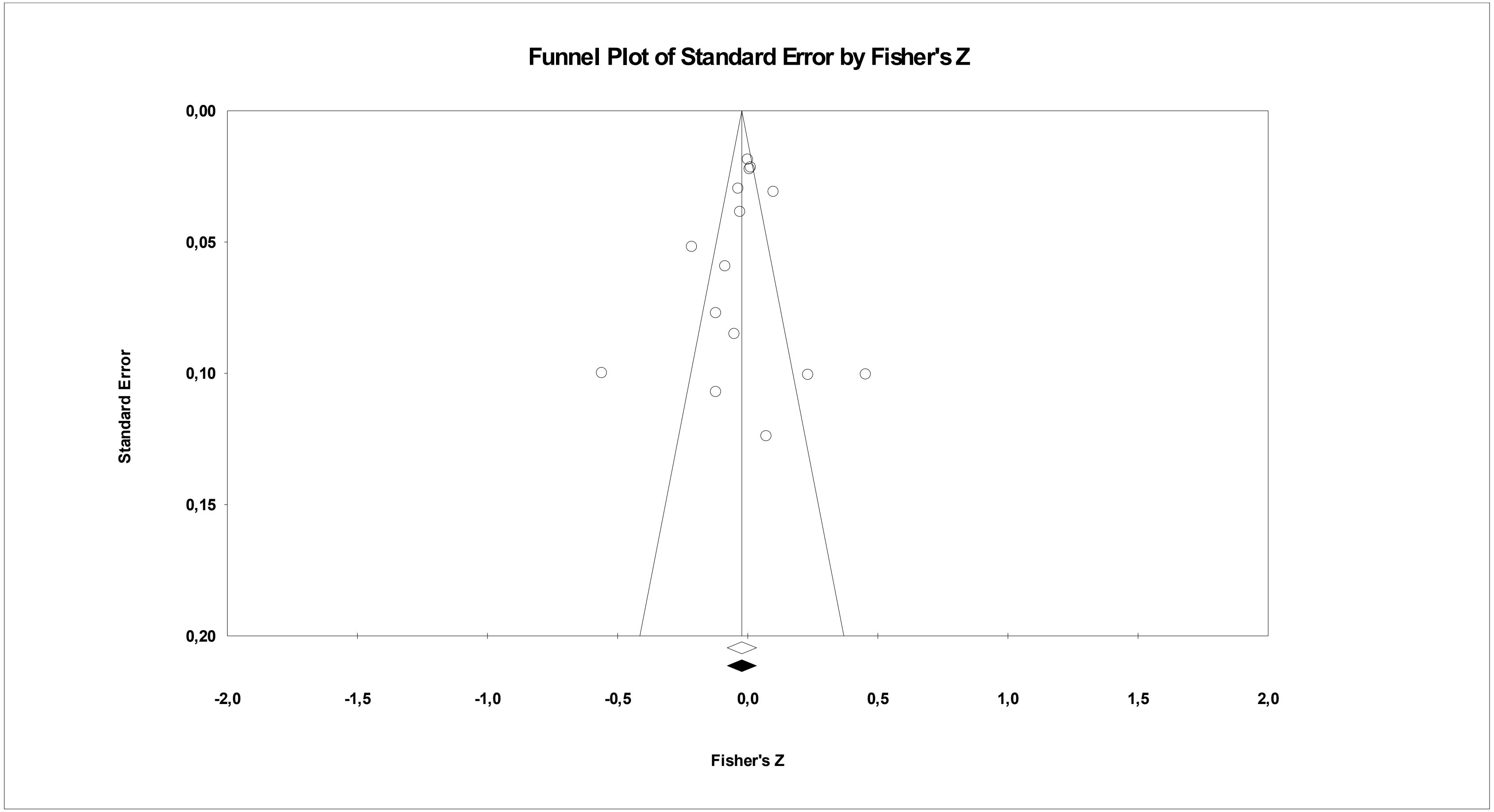

3. Funnel plot: Analysis including subjective health outcomes

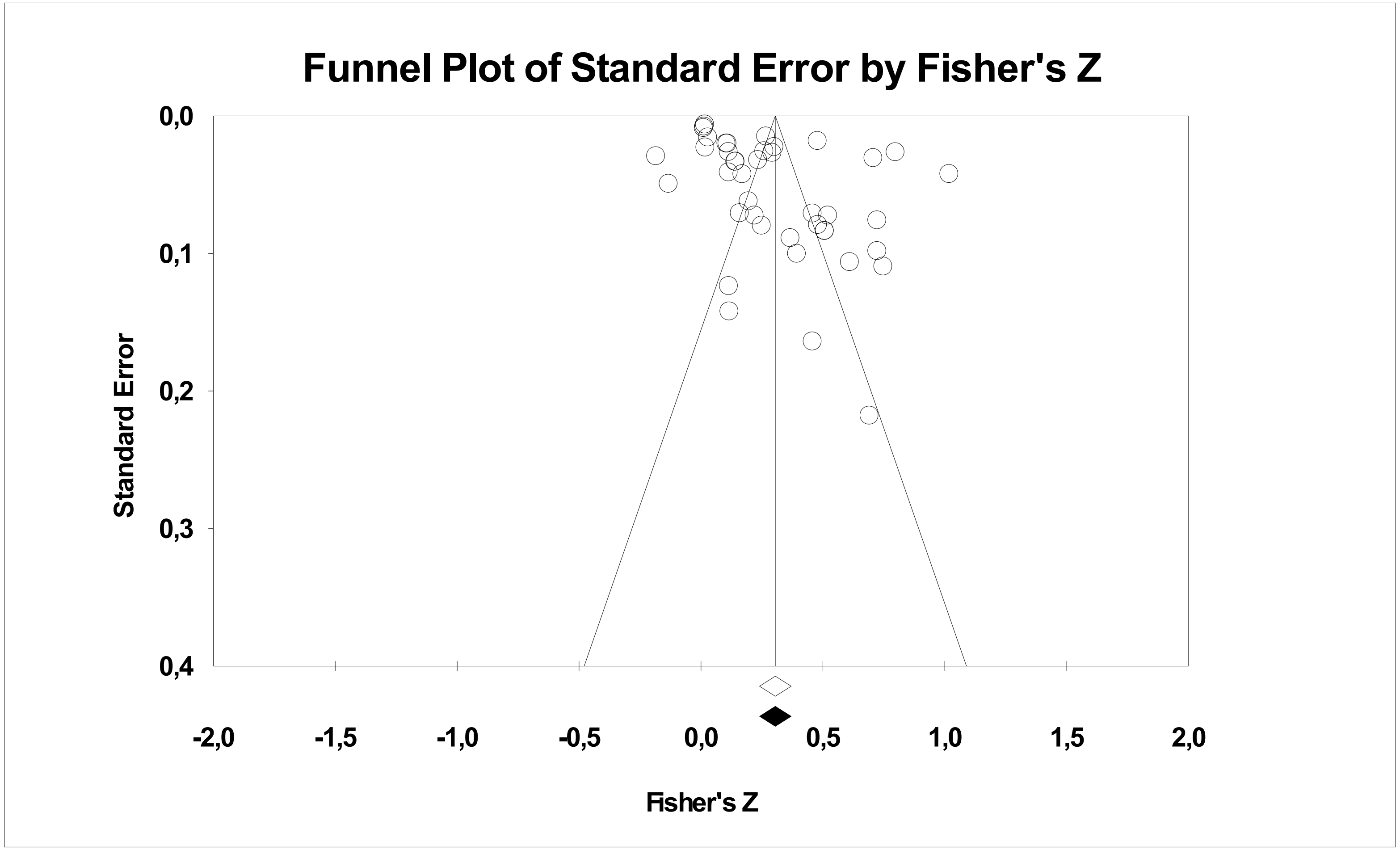

4. Funnel plot: Analysis including behavioural outcomes

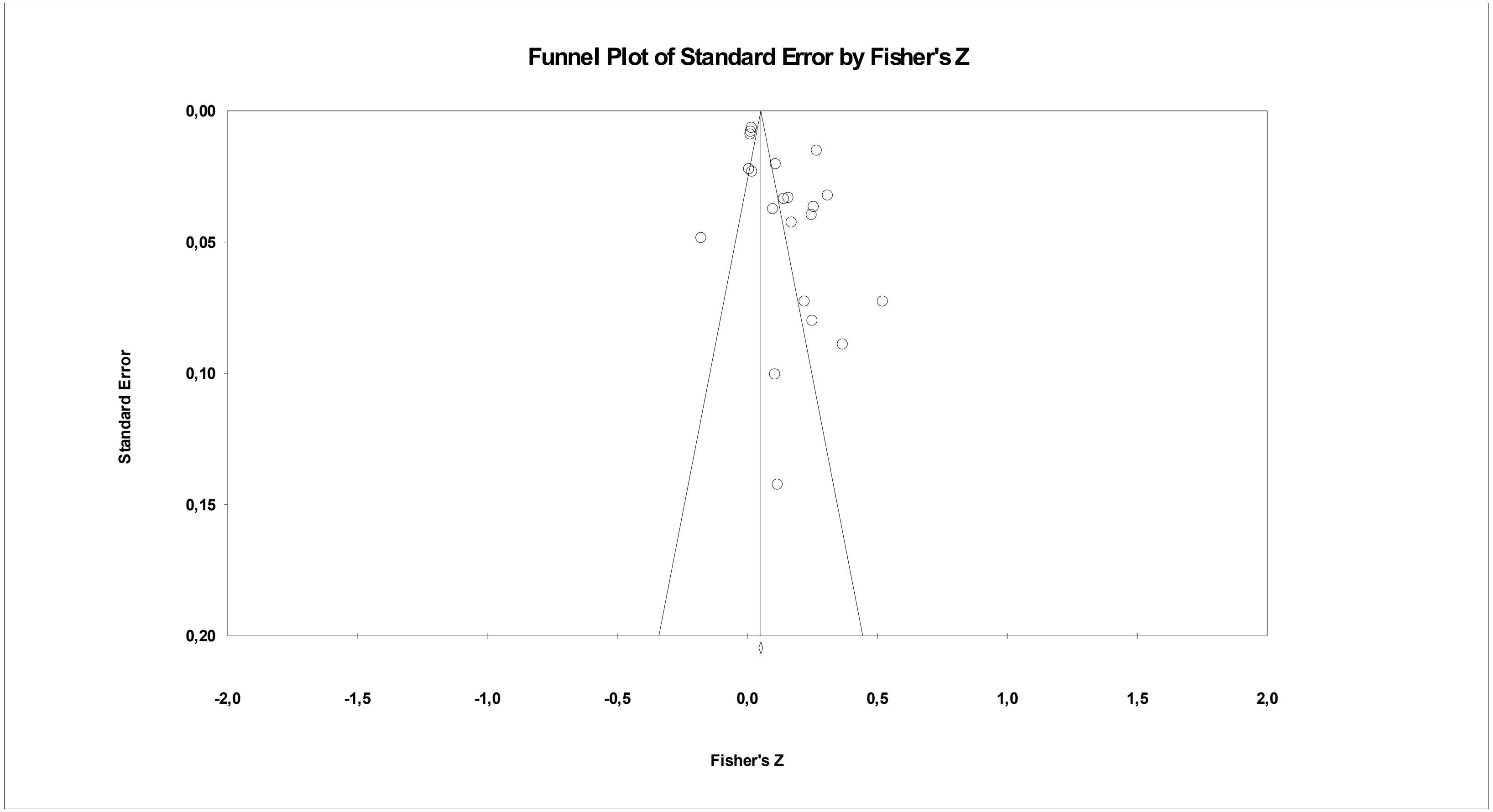

5. Funnel plot: Analysis including subjective experience as outcome

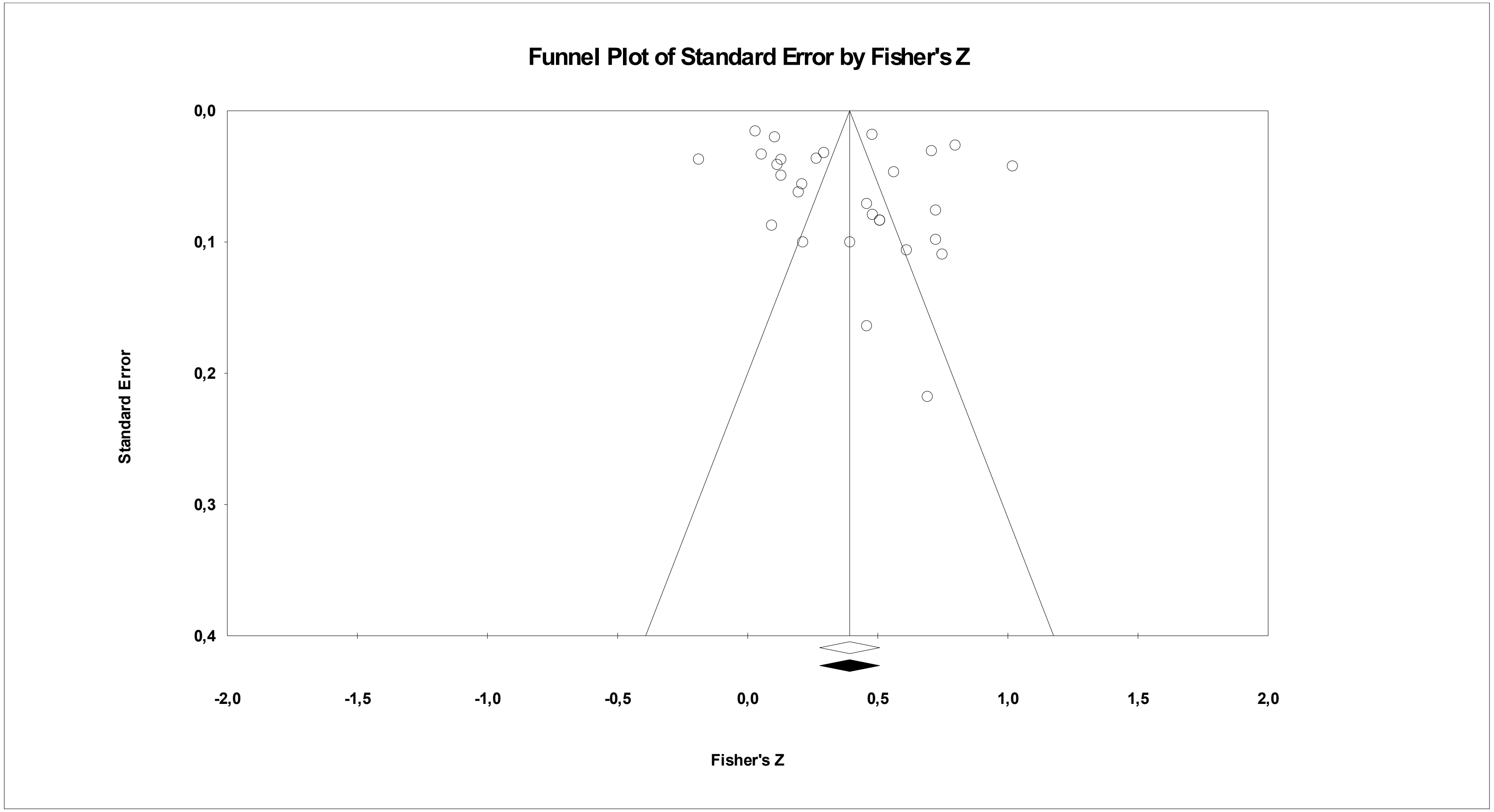

6. Funnel plot: Analysis including patient satisfaction as outcome

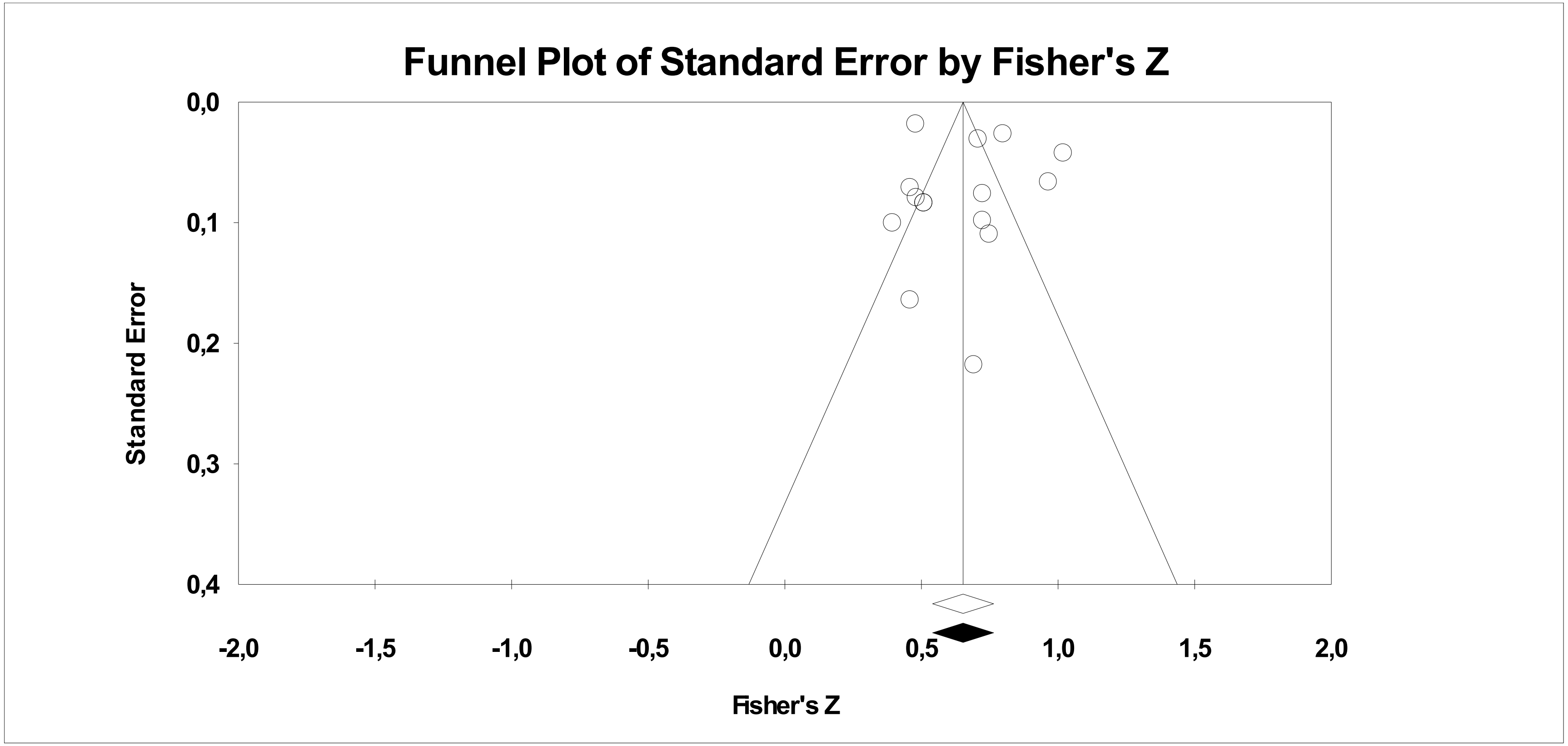

7. Funnel plot: Analysis including health-related quality of life as outcome

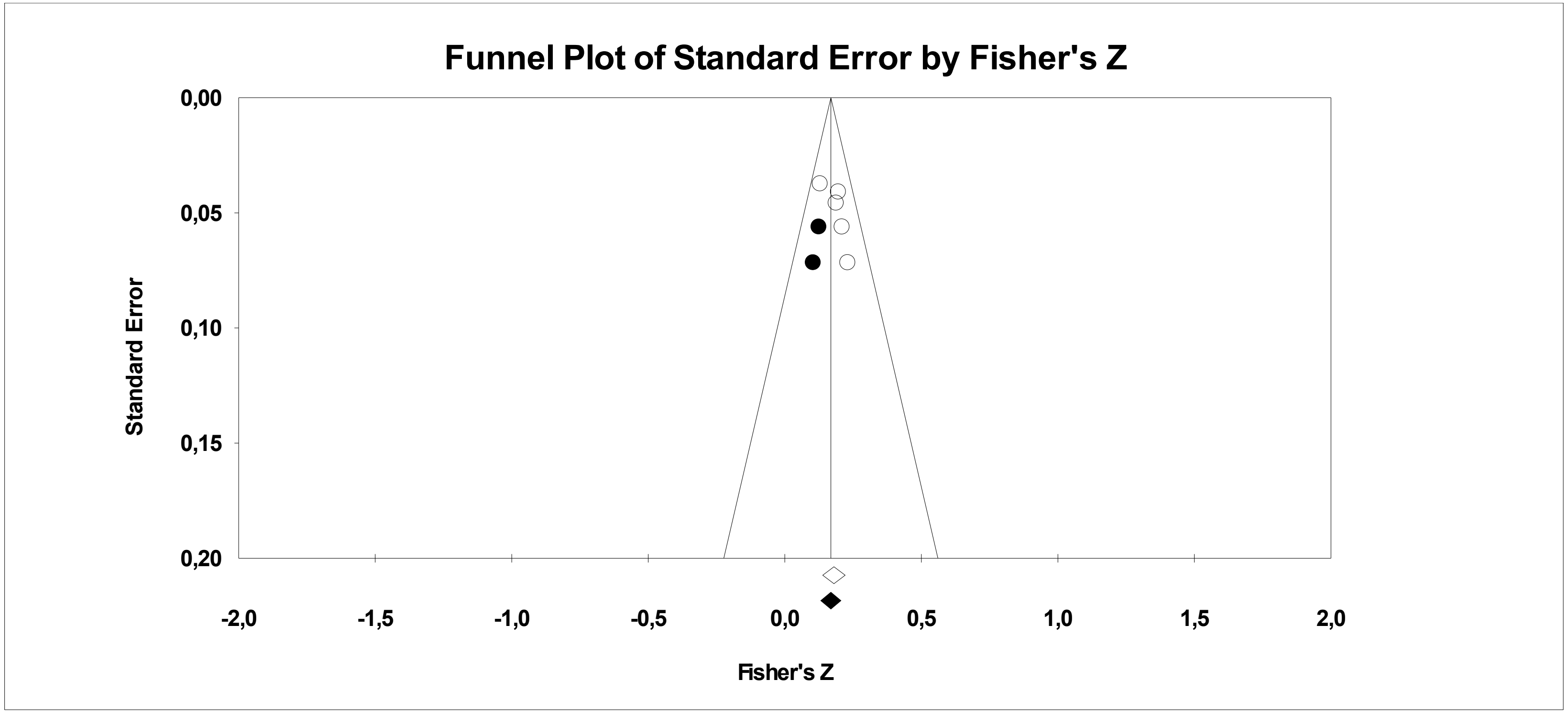

8. Funnel plot: Analysis including symptom-related outcomes

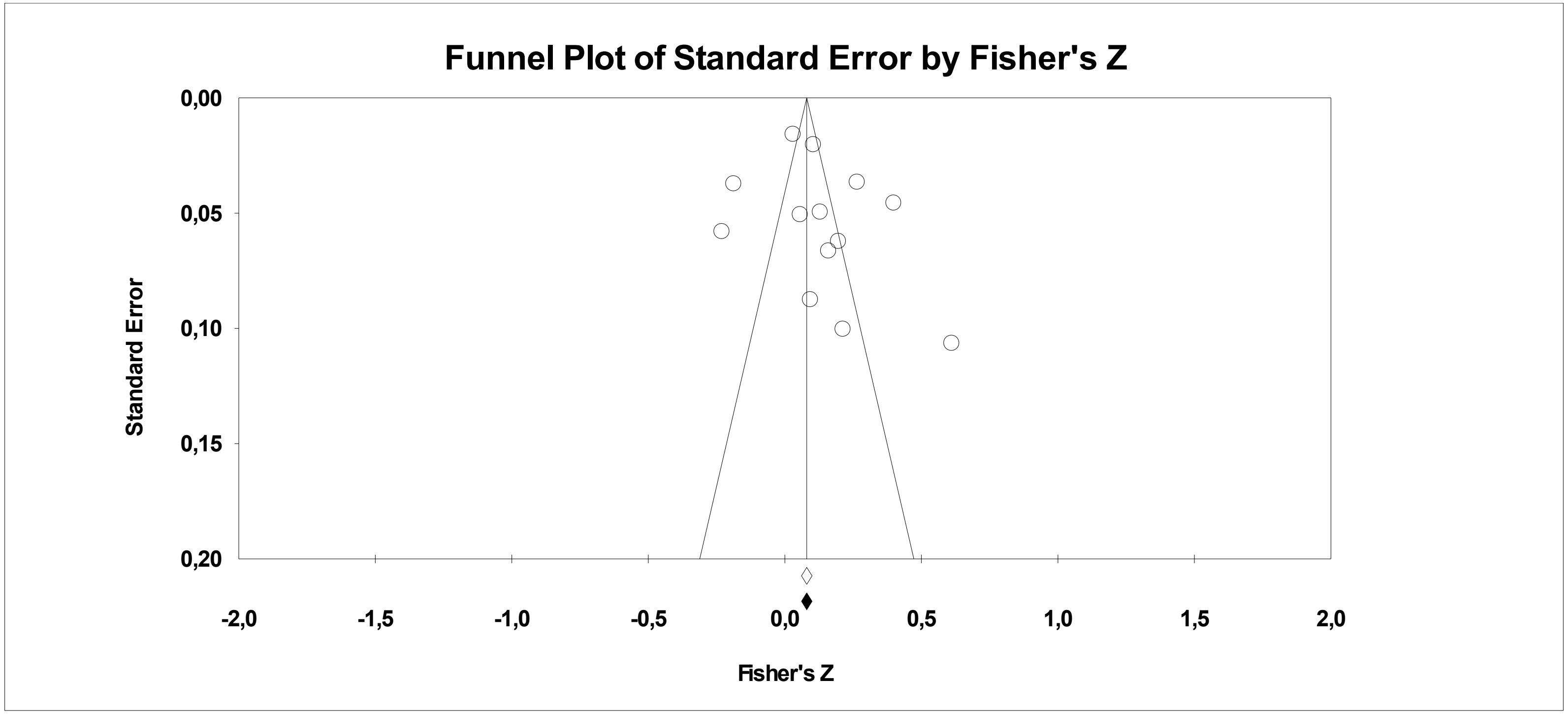

Supplement: S3 File — (PDF) [file pone.0170988.s003.pdf]
