# Supplementary material for: Trust in the health care professional and health outcome: A meta-analysis
Source: PLoS One. 2017 Feb 7;12(2):e0170988. doi: 10.1371/journal.pone.0170988 (PMC5295692; doi:10.1371/journal.pone.0170988)

Supporting File S4. Meta regression.

1. Meta regression for duration of treatment (months) including all studies

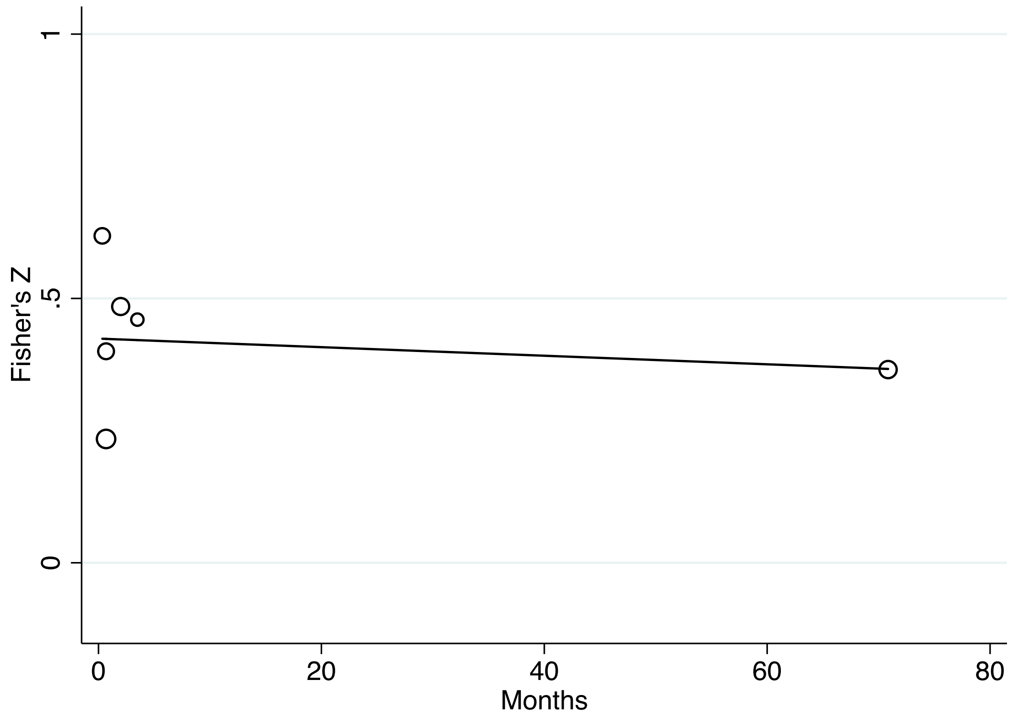

2. Meta regression for duration of treatment (months) excluding outlier study

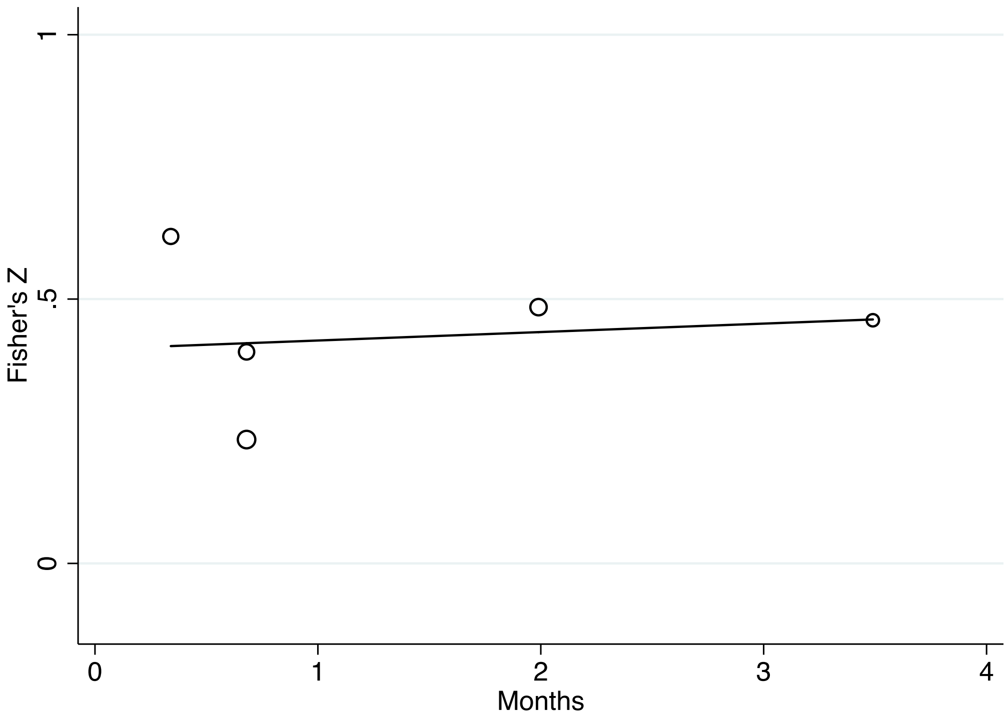

3. Meta regression for study quality including all studies

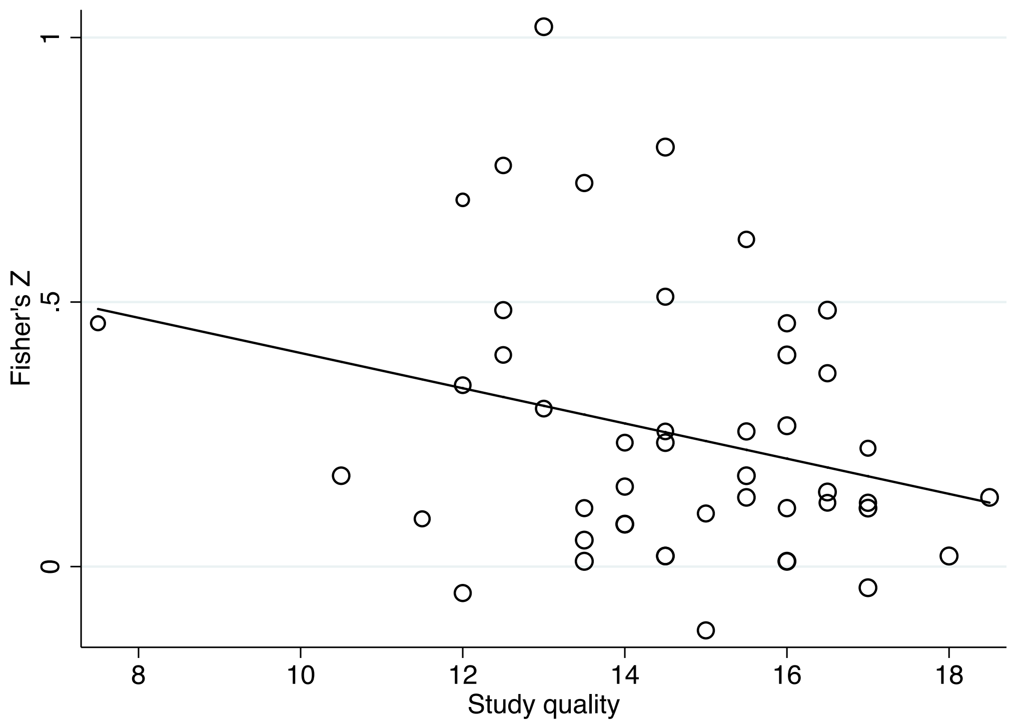

4. Meta regression for study quality excluding outlier study

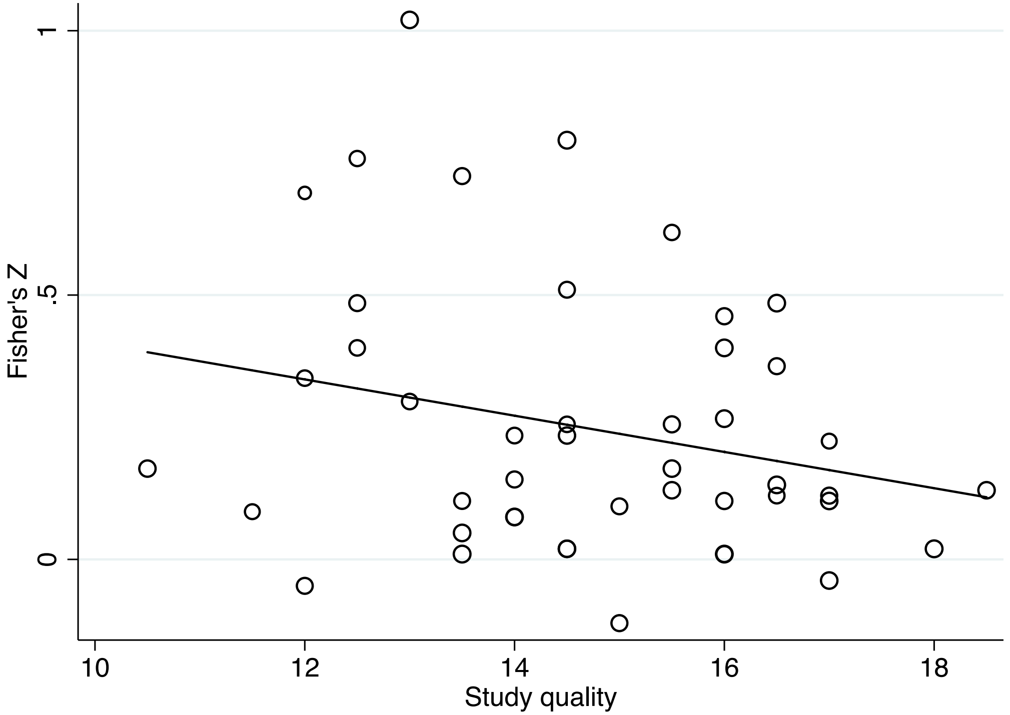

Supplement: S4 File — (PDF) [file pone.0170988.s004.pdf]
