# Supplementary material for: Trust in the health care professional and health outcome: A meta-analysis
Source: PLoS One. 2017 Feb 7;12(2):e0170988. doi: 10.1371/journal.pone.0170988 (PMC5295692; doi:10.1371/journal.pone.0170988)
Supplement: S1 Table — (PDF) [file pone.0170988.s005.pdf]

S1 Table. **Characteristics of Studies Included in the Meta-Analysis**

Author	*Year*	*N*	*Country*	*Disorder*	*Questionnaire*	*STROBE*	*Outcome Measure*
Altice	2001	235	NA	HIV	TiPS	15.5	Acceptance of antiretroviral therapy (ART)
Anderson	1990	177	NA	Diabetes	TiPS	12	Fasting blood glucose level
Anderson*	1990	177	NA	Diabetes	TiPS	12	Fasting blood glucose level; patient satisfaction
Baker	2003	1068[a]	NA	-	TiPS	16.5	General satisfaction,; satisfaction with professional care; satisfaction with depth of relationship; satisfaction with perceived length of consultation
Balkrishnan	2003	2172	NA	Not specified	WFPTS	16	Patient satisfaction with healthcare; physical health
Becker	2008	2224[a]	NA	Diabetes, Healthy, Elevated lipids	TiPS	16	Exercise; diet
Benkert	2006	145	NA	-	TiPS	14.5	Patient satisfaction
Biedrzycki	2011	498	NA	Cancer	PTS	17	Symptom burden; hope; quality of life
Blackstock	2012	187	NA	HIV	WFPTS	16.5	Adherence
Boothroyd	2008	3600	NA	-	TiPS	14.5	General satisfaction
Bosworth	2004	1112	NA	Diabetes, Hypertension, Heart disease	TiPS	16.5	Willingness to undergo surgery
Burge	2009	68	NA	Total knee athroplasty	PONC	11.5	Pain measured by visual analogue scale, functional outcome of the joint athroplasty
Cooper	2009	250	E	Barrett's Oesophagus	TiPS	15	Anxiety; depression; role limitation because of physical problems; role limitation because of emotional problems general perception on health; energy and vitality; social functioning; mental health
Distefano	1981	40	NA	Diagnosis of psychosis, personality disorder	ITS	7.5	Patient satisfaction
Durant	2010	2843	NA	Diabetes, Hypertension, Renal disease	PCAS	16	Blood pressure control
Farin	2010	742	E	Heart disease	TiPS	14.5	Mental health; emotional functioning; social functioning
Fernandez	2012	600	NA	Diabetes	CAHPS-CC	17	Glycosylated hemoglobin, blood lipid cholesterol, systolic blood pressure control

.

Author	*Year*	*N*	*Country*	*Disorder*	*Questionnaire*	*STROBE*	*Outcome Measure*
Freburger	2003	713[a]	NA	Rheumatic disease	TiPS	13.5	Diagnosis of fibromyalgia, osteoarthritis, rheumatoid arthritis
Garcia-Gonzalez	2009	200	NA	Rheumatic disease	TiPS	16	Patient satisfaction with clinical visits
Graham	2013	188	NA	HIV	WFPTS	15	Early diagnosis of HIV: CD4 cell counts
Gregg	2010	11 928	NA	Diabetes	PCAS	18	Probability of missing preventative care
Guipponi	2009	161	E	Diagnosis of Affective disorder, Schizophrenia, Addiction, Other	ICS	12.5	Patient satisfaction
Guipponi*	2009	161	E	Diagnosis of Affective disorder, Schizophrenia, Addiction, Other	ICS	12.5	Patient satisfaction
Hillen	2013	175[a]	Au	Cancer	TiOS	13.5	Satisfaction with the oncologist
Hunfeld	1999	24[a]	E	Fetal Anomaly (pregnant women)	CPQ	12	Satisfaction with prenatal consultation
Kao	2012	192[a]	A	Diagnosis of Mood disorder, Schizophrenia	ICS	14	Diagnosis schizophrenia, mood disorder; adherence
Kerse	2004	490	Au	-	TiPS	14.5	Medication compliance
Lee	2009	480[a]	A	Diabetes	TiPS	18.5	Health-related quality of Life; BMI, glycosylated hemoglobin, blood lipid control; complications self-efficacy; outcome expectation; adherence
Mainous	2004	119	NA	Cancer	TiPS	14	Earlier detection of cancer: Stage of cancer
Mancuso	2010	102	NA	Diabetes	HCR	14.5	Glycosylated hemoglobin con-centration; self-care activities; depression
Moffet	2011	186 306	NA	Diabetes	TiPS	13.5	Lab attendance for control of glycosolated haemoglobin, blood lipid, serum creatinine, urinary albumin
Ngyuen	2009	235[a]	NA	Inflammatory Bowel Disease	TiPS	16	Self-reported non adherence, overall adherence, medication adherence
Ostertag	2013	374	NA	HIV, Other	TiPS	10.5	Diagnosis of HIV; depression; health care utilization, self-efficacy

Author	*Year*	*N*	*Country*	*Disorder*	*Questionnaire*	*STROBE*	*Outcome Measure*
Peters	2007	145	-	Hypertension	TiPS	13.5	Diastolic and systolic blood pressure level; satisfaction with care
Piette	2005	912[a]	NA	Diabetes	PCAS	14	Non-cost related underuse; depressive symptoms; comorbidities
Platonova	2008	554	NA	-	TiPS	13	Patient satisfaction with physician service
Rosman	2012	162	-	-	TEPQ	16.5	Non-adherence
Sizer	2008	86[a]	-	-	TiPS	12.5	Patient satisfaction
Slean	2012	502[a]	-	Diabetes	CAHPS-CC[b]	17	Emotional and mental distress associated with management of diabetes
Spain	2008	1054	NA	Cancer	PCAS[b]	14.5	Personal responsibility for prostate care; cancer is diagnosed symptomatically (later diagnosis)
Taha	2011	347	NA	Cancer, Healthy	TiPS	14	Depressive symptoms; medical decision regret
Thüm	2012	130	E	Severely injured	CPQ	15.5	Experienced support by the physician
Trachtenberg	2005	2172	NA	-	WFPTSb	15.5	Adherence
Tucker	2011	229	NA	Diabetes, Hypertension	TiPS	13	Patients interpersonal control; patient satisfaction; physical stress; health promoting lifestyle; diet adherence; medication adherence
Vina	2012	182[a]	NA	Lupus erythematodes	WFPTS	16	Willingness to take immunosuppressive medication
Vissman	2013	91	NA	HIV	WFPTS	17	Adherence
Waters	2010	1572	NA	Cancer	TiPS	15.5	Perceived risk of recurrence

Note. Study Quality was assessed using the STROBE Statement-checklist of items that should be included in reports of observational studies: A value of < 10 is considered as poor study quality; N=total sample size of the study; NA=North America; E=Europe; A=Asia; Au=Australia; -=not specified; Other=a group of participants is not specified with regard to disorder; TiPS=Trust in Physician Scale; WFPTS=Wake Forest Trust Scale; PCAS=Trust Scale of the Primary Care Assessment Survey; CAHPS-CC=Trust Scale of Consumer Assessment of Healthcare Providers and Systems Cultural Competence; ICS= Trust Scale of the Illness Concept Scale; TiOS=Trust in Oncologist Scale; CPQ=Trust Scale of the Cologne Patient Questionnaire; HCR=The Health Care Relationship Trust Scale; PTS=Patient Trust Scale; ITS=Interpersonal Trust Scale; TEPQ=Trust in the Emergency Department Questionnaire; PONC=Patient's Opinion of Nursing Care; BMI=Body mass index.
*Described as a second independent study population in article, therefore separately included in analysis.
[a] Descriptive N was used, since total N was missing.
